# Cloud–edge–device collaborative computing in smart agriculture: architectures, applications, and future perspectives

**DOI:** 10.3389/fpls.2025.1668545

**Published:** 2025-10-14

**Authors:** Pengpeng Yu, Fei Teng, Wenhui Zhu, Chaoping Shen, Zhenping Chen, Jinxiu Song

**Affiliations:** ^1^ School of Agricultural Engineering, Jiangsu University, Zhenjiang, China; ^2^ School of Finance and Economics, Jiangsu University, Zhenjiang, China; ^3^ Tianshui Huatian Technology Co., Ltd., Tianshui, Gansu, China; ^4^ School of Energy and Power Engineering, Jiangsu University, Zhenjiang, China; ^5^ School of Electronic and Information Engineering, Suzhou University of Science and Technology, Suzhou, China

**Keywords:** smart agriculture, cloud–edge–device collaboration, distributed computing, edge artificial intelligence (edge AI), environmental monitoring, intelligent irrigation, precision livestock farming, pest and disease management

## Abstract

Smart agriculture is rapidly evolving in response to growing global demands for food security and sustainable resource management. Cloud–edge–device collaborative computing has emerged as a transformative paradigm, addressing the limitations of traditional centralized architectures by enabling distributed intelligence, real-time processing, and adaptive decision-making. This review provides a comprehensive overview of the architectures, technical characteristics, and application scenarios of cloud–edge–device collaboration in agriculture. Key domains covered include environmental monitoring, intelligent irrigation, UAV–machinery coordination, livestock health management, and pest and disease control. Major challenges such as device heterogeneity, data consistency, resource constraints, and privacy concerns are identified and discussed. Furthermore, six critical research directions are outlined, including intelligent scheduling algorithms, lightweight edge AI, hierarchical data fusion, federated learning, interoperability frameworks, and digital twin technologies. This review aims to serve as a practical reference and theoretical foundation for advancing the design and implementation of next-generation smart agriculture systems.

## 1 Introduction

Agriculture, as the foundation of human survival and social development, is currently facing unprecedented challenges. On one hand, the global population continues to grow rapidly, leading to soaring food demand. On the other hand, the sector is constrained by shrinking arable land, water scarcity, climate change, land degradation, and the frequent occurrence of pests and diseases, all of which severely limit productivity. As traditional farming methods struggle to meet modern agricultural demands, there is an urgent need to leverage advanced information technologies to promote digital and intelligent transformation toward high-quality and sustainable agricultural development (Ali Lakhiar, 2020; He et al., 2020; Sarkar et al., 2022; Wang X. et al., 2024; Iqbal et al., 2025).

In recent years, the emergence and rapid development of technologies such as Artificial Intelligence (AI), Big Data, and the Internet of Things (IoT) have led to the rise of Smart Agriculture, a paradigm that emphasizes data-driven operations, real-time sensing, and intelligent decision-making (Yang et al., 2022; Zhang et al., 2022a; Wang W. et al., 2024; Yuanyuan, 2024; Zhu et al., 2024). By constructing integrated sensing systems and intelligent control frameworks, Smart Agriculture enhances automation, precision, and fine-grained management in agricultural production. However, the agricultural environment is characterized by wide geographic distribution, scattered nodes, complex conditions, and unstable network connectivity, which pose significant challenges to system responsiveness, computing capacity, and energy efficiency. Traditional centralized cloud-based models are often insufficient for such scenarios due to high latency, bandwidth limitations, and poor adaptability (Li et al., 2020a; Akbari et al., 2024).

In this context, Cloud-Edge-Device Collaborative Computing has emerged as a new distributed intelligent architecture, offering a promising technical foundation for Smart Agriculture. Based on a multi-tier design principle, it integrates cloud computing, edge computing, and end devices to form an intelligent closed-loop system that spans from front-end sensing to mid-tier processing and back-end decision-making (Li et al., 2020b; Akbari et al., 2023).

Cloud computing provides centralized processing power and storage for large-scale agricultural data analysis, deep learning, and historical optimization (Fatouros et al., 2023). Edge computing enables low-latency data processing and local control by deploying computing nodes near the data source, such as gateways or edge servers in fields or greenhouses (Gu H. et al., 2024). Device-side computing (end computing) focuses on real-time data collection and basic logic operations via sensors, cameras, unmanned aerial vehicles (UAVs), and smart agricultural machinery. The collaborative operation of these three layers significantly alleviates the burden on central cloud servers, improves system responsiveness and fault tolerance, and enhances the autonomous capabilities of field-level agricultural systems. This architecture is particularly suited for scenarios requiring real-time decision-making and resilience in environments with unreliable connectivity, such as open-field farming, greenhouse management, and livestock monitoring (Kalyani et al., 2024).

This paper aims to provide a comprehensive review of the typical applications and recent advancements in cloud-edge-device collaborative computing within the context of smart agriculture. It identifies the key challenges and technical bottlenecks currently faced in this field and offers a forward-looking perspective on potential development directions. While existing studies have predominantly focused on the individual application of either cloud computing or edge computing in agricultural scenarios, in-depth investigations into the holistic cloud-edge-device collaborative architecture remain limited. This is particularly true in agricultural environments characterized by fragmented data sources, constrained edge resources, and heterogeneous end devices—conditions that demand specialized coordination models and deployment strategies. Therefore, this review seeks to address this critical research gap and provide both theoretical insights and practical guidance for the future design and optimization of intelligent agricultural systems.

Cloud–edge–device collaborative computing has emerged as a multi-tier distributed architecture that integrates end-device perception, edge-level real-time processing, and cloud-based large-scale decision-making. This framework addresses latency, bandwidth and resilience challenges in agriculture by enabling localized inference at the device/edge layers while leveraging the cloud for heavy analytics and cross-regional coordination. To provide a structured overview, this paper is organized as follows: Section 2 analyzes the technical characteristics and architectural models of cloud–edge–device collaboration in smart agriculture. Section 3 discusses key technical challenges associated with multi-level computing deployment. Section 4 presents representative application scenarios, including environmental monitoring, irrigation control, UAV coordination, livestock health monitoring, and pest and disease management. Section 5 outlines emerging trends and future research directions. Section 6 concludes the review with key insights and recommendations.

## 2 Cloud–edge–device coordination framework: technical characteristics and architectures

### 2.1 Technical characteristics and functional division of cloud, edge, and end device computing

In smart agriculture systems, data collection, transmission, processing, and decision-making require coordinated operations across multiple computing layers. Cloud computing, edge computing, and device (or end) computing each play distinct roles, together forming a distributed intelligent computing architecture tailored to agricultural environments (Fatouros et al., 2023; Gu H. et al., 2024).

**Figure 1:**
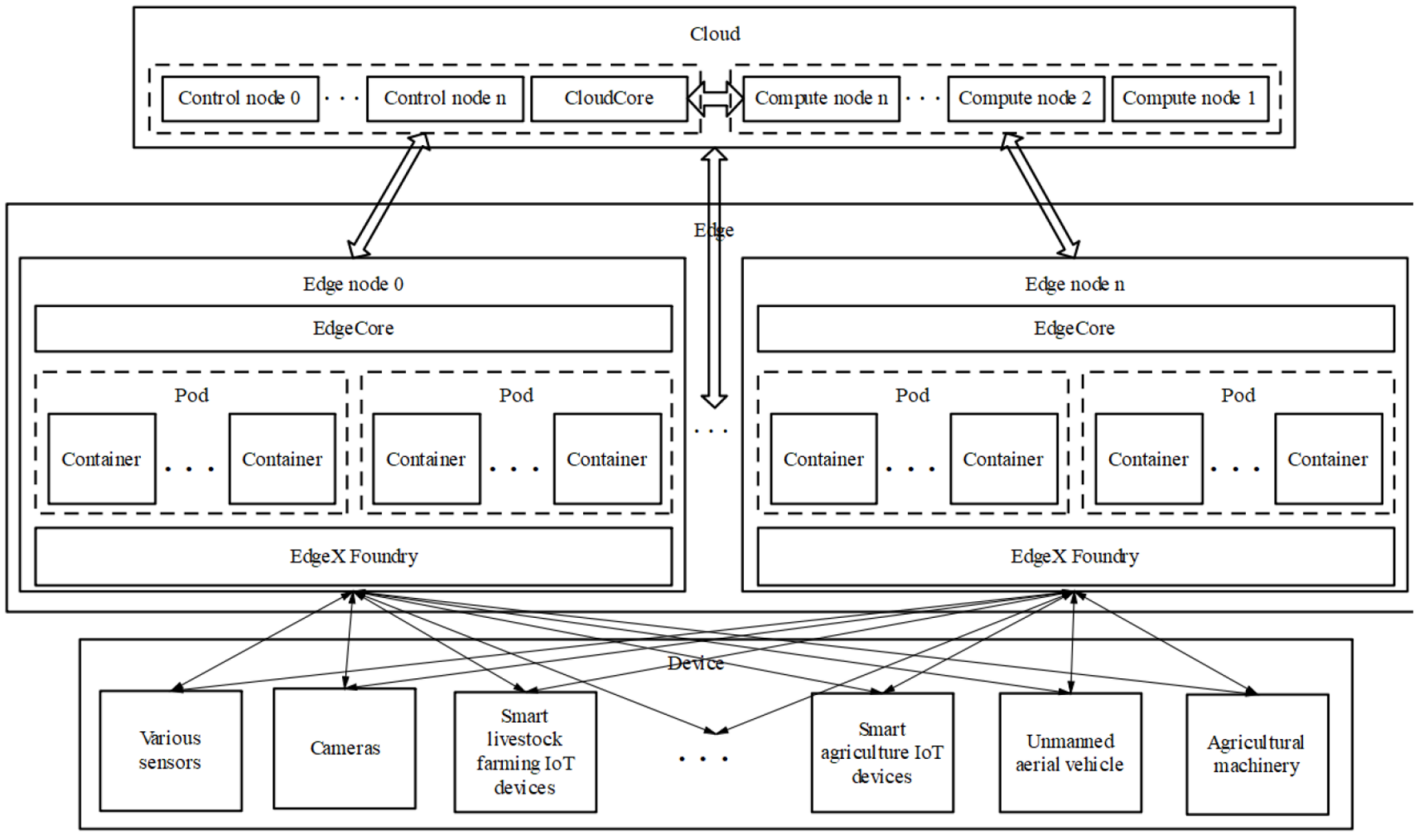
Edge computing system logic diagram.

**Figure 1** illustrates the logical architecture of the edge computing system, which depicts in detail the data flow and interaction between end devices, edge nodes, and the cloud computing center. Through this diagram, It is evident that data is collected from end devices, undergoes preliminary processing at edge nodes, and is then transmitted to the cloud computing center for further analysis. Building on the architecture defined in Section 1 and illustrated in **Figure 1**, this section details the technical characteristics and functional division of each layer (device, edge, cloud), focusing on hardware constraints, communication protocols, and computational trade-offs that shape agricultural deployments.

1. Cloud Computing is primarily responsible for the centralized processing and storage of large-scale agricultural data. With its robust computational power, abundant storage capacity, and high resource elasticity, cloud computing is well-suited for tasks such as model training, cross-regional data integration, and historical trend analysis. In smart agriculture, cloud platforms often support macro-level decision-making functions, including pest and disease forecasting, climate simulation, and crop growth modeling (Tripathy et al., 2022; Morales-García et al., 2023).
2. Edge Computing operates at local nodes near data sources, such as agricultural gateways, edge servers, and onboard terminals in farming equipment. Its core strengths lie in low latency, real-time responsiveness, and bandwidth efficiency. This makes it particularly effective in scenarios requiring immediate processing and localized decision-making, such as real-time video analytics, emergency event handling, and automatic control systems. Examples include the autonomous regulation of greenhouse environments and responsive irrigation control based on field conditions, both of which can be managed efficiently through edge nodes (Ayoade et al., 2023; Ariss et al., 2024).
3. Device or End Computing represents the layer closest to the physical sensing environment. It includes a wide range of agricultural IoT devices such as sensors, cameras, unmanned aerial vehicles (UAVs), and agricultural robots. These devices are responsible for front-line data acquisition and basic preprocessing tasks, such as capturing crop images or monitoring environmental parameters. They also enable simple, real-time decision functions like threshold-based alerts or actuator control (Jaiswal and Rawat, 2021; Li X. et al., 2023).

Together, these three layers form a complementary, hierarchical, and collaborative computing model: devices serve as the “perception” layer, edges as the “analysis” layer, and the cloud as the “aggregation and decision-making” layer. This multilayer synergy enables the seamless execution of intelligent agricultural operations and supports the evolution toward highly automated and data-driven farming systems.

### 2.2 Typical architectural models of cloud-edge-device collaboration

In practical deployments, cloud-edge-device collaboration can be structured into various architectural models depending on task complexity, communication capabilities, and resource constraints. The most commonly adopted architectures include the following:

1. Hierarchical Architecture: In cloud-edge-device collaborative systems, the hierarchical architecture is the most commonly adopted structural model. This architecture divides the system into three layers: the perception layer, the processing layer, and the decision-making layer, which correspond to end devices, edge nodes, and the cloud computing center, respectively. Together, they form a tiered system characterized by a “perception–processing–decision” workflow (Rojas et al., 2022; Penzotti et al., 2024).

**Figure 2:**
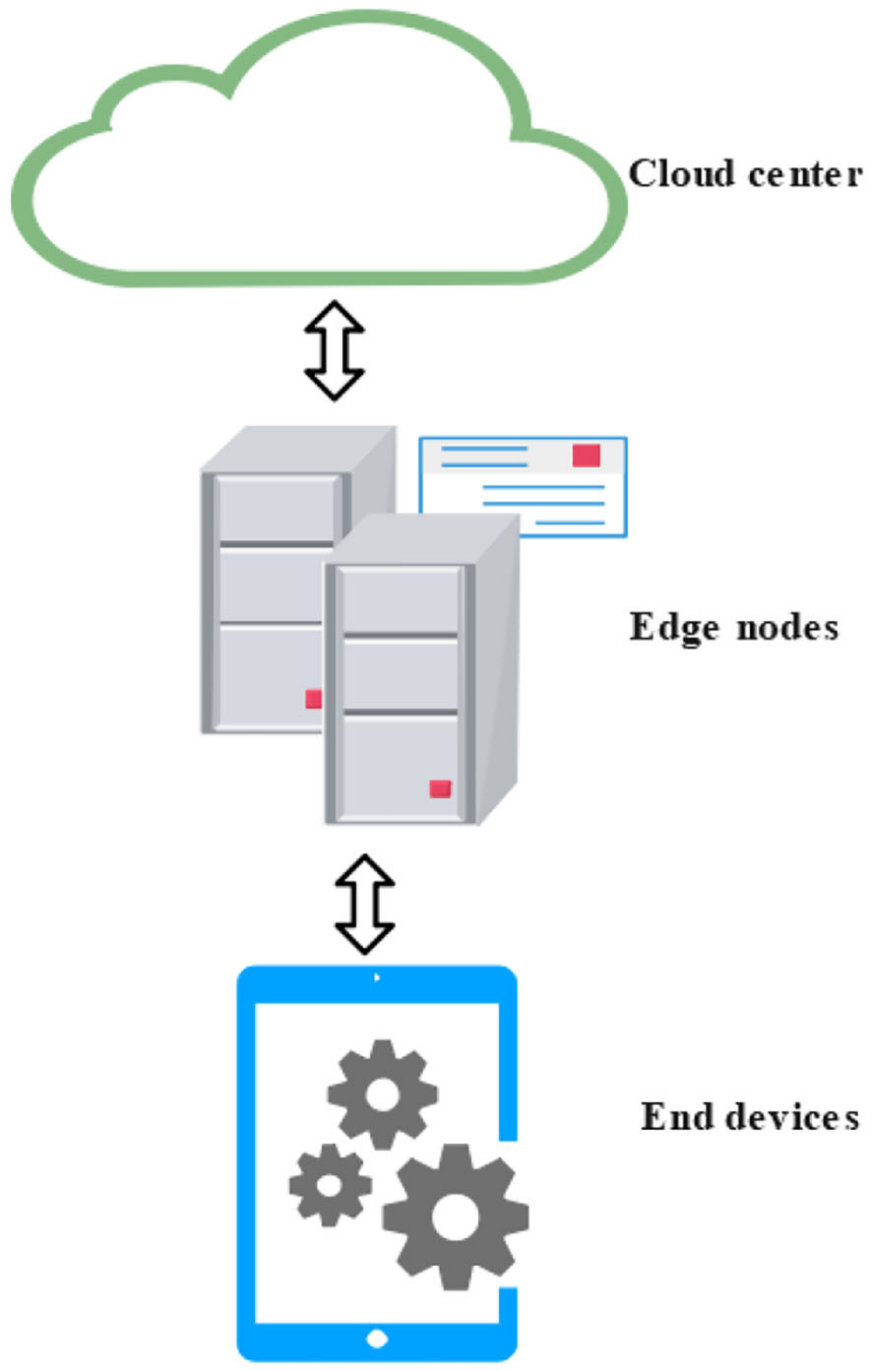
Hierarchical cloud-edge-device collaborative architecture.

As illustrated in **Figure 2**, data is first collected by end devices at the perception layer and then transmitted upward to edge nodes, where preliminary processing is performed. Subsequently, the processed data is sent to the cloud center for more complex analysis and final decision-making. This layered model is particularly well-suited for application scenarios where task complexity increases progressively across layers—such as in agricultural environmental monitoring and precision farming. By distributing computational tasks across multiple tiers, the hierarchical architecture enables more efficient resource allocation and significantly improves the overall performance and responsiveness of the system.

2. Star-like Architecture: The star-like architecture is a cloud-edge-device collaborative model that centers on the cloud computing platform as the core coordination and communication hub. In this architecture, all edge nodes and end devices are connected through a central node—typically the cloud—which is responsible for task scheduling, data aggregation, and overall coordination (Yadav and Kumar, 2023; Wu et al., 2025).

**Figure 3:**
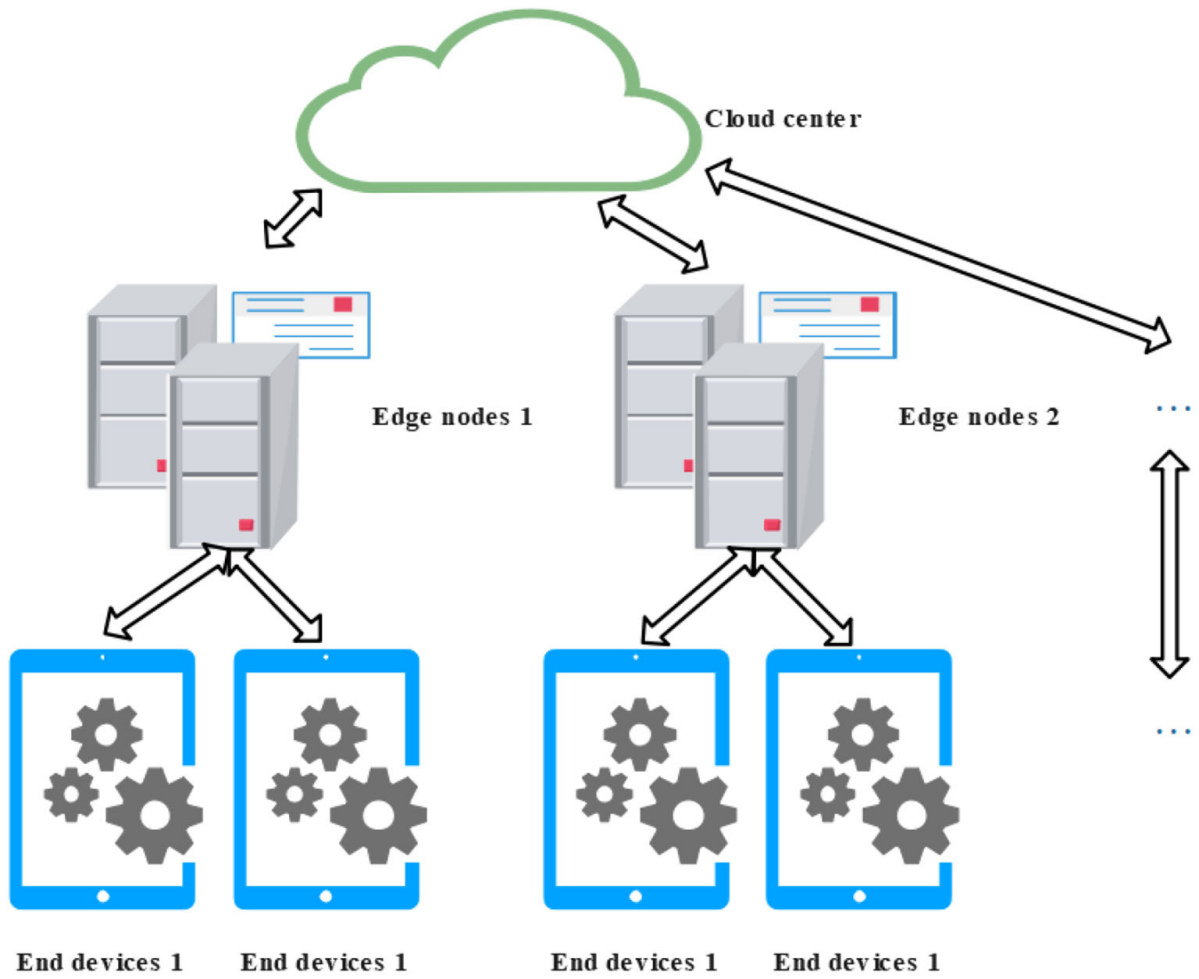
Star-like cloud-edge-device collaborative architecture.

As depicted in **Figure 3**, the cloud platform serves as the central controller, managing the distribution of tasks and the collection of data, while edge nodes and end devices are arranged around it in a star-shaped topology. Communication and task execution are achieved via the central cloud node, enabling unified control across a distributed system. This architectural model is particularly well-suited for scenarios involving centralized task coordination and requiring high deployment flexibility, such as agricultural drone fleet management or remote pest and disease monitoring systems. It leverages the centralized computing and orchestration capabilities of the cloud to enhance the scalability, adaptability, and ease of deployment of smart agricultural applications.

3. Chain or Relay Architecture: The chain (or relay) architecture is a cloud-edge-device collaborative model in which data is transmitted sequentially along a chained path. In this architecture, data originates from end devices and is relayed through a series of edge nodes, each performing intermediate processing, before finally reaching the cloud computing center (Alonso et al., 2020; Debauche et al., 2022; Kum et al., 2023).

**Figure 4:**
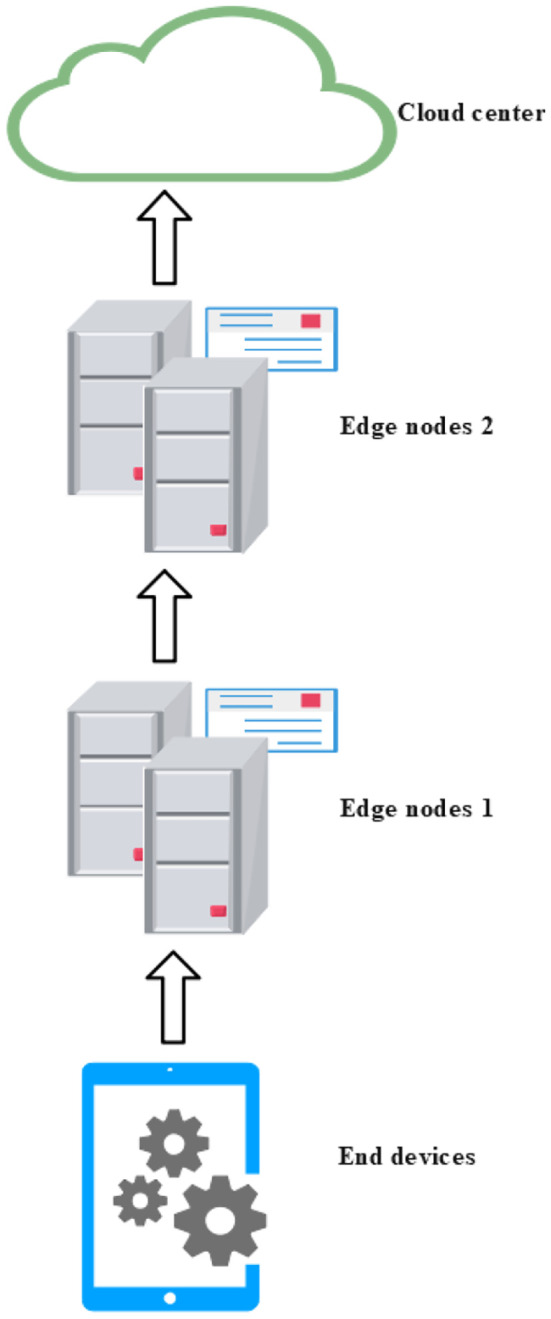
Chain (relay) cloud-edge-device collaborative architecture.

As illustrated in **Figure 4**, this architectural pattern is particularly suitable for regions with limited bandwidth or constrained computational resources at the edge or device level, such as agricultural monitoring systems in remote or mountainous areas. By utilizing relay edge nodes for data aggregation and forwarding, the chain architecture effectively alleviates transmission bottlenecks and enhances the reliability and stability of long-distance data communication in resource-constrained environments.

These architectural models can be flexibly combined and customized to fit the unique requirements of different agricultural scenarios. By adapting deployment strategies to specific operational conditions, it is possible to enhance the overall efficiency, responsiveness, and reliability of smart agriculture systems.

### 2.3 Technical challenges and key issues

Despite the promising potential of cloud-edge-device collaboration in smart agriculture, its deployment and real-world implementation still face a number of significant challenges, including the following:

1. Complexity of Managing Heterogeneous Devices: The agricultural environment involves a wide variety of devices—such as sensors, cameras, and farming machinery—from different manufacturers, often using incompatible protocols. This diversity presents considerable difficulties in device integration, coordination, and unified management (Ju and Son, 2020).
2. Data Consistency and Synchronization: Ensuring the consistency, timeliness, and security of data across multiple distributed nodes—particularly between edge devices and the cloud—remains a critical technical challenge. The problem becomes more pronounced in scenarios involving network disconnections, latency, or data conflicts (Li et al., 2025; Prymushko et al., 2025).
3. Bottlenecks in Real-Time Response and Task Scheduling: Certain agricultural tasks, such as emergency pest control or intelligent irrigation, require strict real-time responsiveness. Inadequate task scheduling mechanisms may lead to high latency or delayed responses, thereby compromising operational efficiency and production safety (Liang and Fan, 2021; Bachelet et al., 2022).
4. Energy Consumption and Computational Resource Trade-offs: Edge and end devices are often constrained by limited power supply and computing capacity. Achieving intelligent processing while maintaining low energy consumption is a major concern, particularly in off-grid environments or battery-powered deployments (Alghazali et al., 2025; Aquino-Brítez et al., 2025).
5. Security and Privacy Protection: Agricultural data typically carries strong spatiotemporal attributes. If compromised, it could expose sensitive information about cultivation activities or production processes. This necessitates the implementation of robust communication encryption, data anonymization, and fine-grained access control mechanisms (Yang et al., 2021; Shen, 2023).

Addressing these challenges requires ongoing research and innovation in areas such as hardware–software co-design, system architecture optimization, task scheduling algorithms, communication protocols, and data security frameworks. Advancements in these domains will be essential to achieving efficient, secure, and scalable adoption of cloud-edge-device collaborative technologies in smart agriculture.

## 3 Key technical requirements and challenges in smart agriculture

### 3.1 Key characteristics of agricultural application scenarios

Smart agriculture differs fundamentally from industrial manufacturing or urban Internet of Things (IoT) environments in terms of spatial distribution, environmental conditions, and communication infrastructure. These differences give rise to a number of unique challenges that must be addressed when designing intelligent agricultural systems. Specifically, the following key characteristics define agricultural scenarios:

1. Widely Distributed and Heterogeneous Data Sources: Agricultural operations are typically carried out across vast, open, and geographically dispersed environments. Sensors, cameras, agricultural machinery, and other edge devices are deployed in large quantities and across varied terrains. These devices often differ significantly in type, manufacturer, and communication protocol, resulting in a highly heterogeneous and decentralized data ecosystem (Sun et al., 2022; Gu W. et al., 2024; Xu et al., 2024).
2. High Demand for Real-Time Local Responsiveness: Many agricultural events—such as abrupt weather changes, pest outbreaks, or sudden shifts in soil moisture levels—exhibit unpredictability and rapid dynamics. Systems deployed in such environments must be capable of fast, localized decision-making to mitigate risks in a timely manner. Relying solely on cloud-based processing may introduce unacceptable latency, potentially leading to delayed responses and losses in yield or quality (Ji et al., 2021; Liu et al., 2023; Ma et al., 2024).
3. Weak Network Infrastructure: In many rural and remote agricultural regions, communication infrastructure is either underdeveloped or unreliable. Network coverage is often intermittent, and available bandwidth is limited. These constraints make it difficult to implement traditional centralized cloud computing solutions that require stable, high-throughput connectivity for continuous data transmission and remote control (Tao and Wei, 2024; Zhang T. et al., 2024; Zhao et al., 2024).
4. Energy Sensitivity and Resource Constraints: A significant portion of agricultural IoT devices are low-power embedded systems that depend on limited energy sources, such as batteries or solar panels. These systems are particularly sensitive to power consumption and have constrained processing and memory resources. This necessitates the adoption of lightweight computing frameworks, efficient communication protocols, and intelligent energy-aware scheduling strategies (Niu et al., 2025).

These distinctive characteristics demand that agricultural information systems be highly robust, autonomous, and scalable. The conventional centralized cloud-centric architecture is increasingly inadequate in meeting the demands of such dynamic and resource-constrained environments. As a result, cloud-edge-device collaborative computing has emerged as a key enabling paradigm for the advancement of smart agriculture, offering a more distributed, resilient, and adaptive computational framework tailored to real-world agricultural applications.

### 3.2 Mapping smart agriculture needs to cloud-edge-device collaboration

Facing the above scenario characteristics, the cloud-edge-device architecture demonstrates strong adaptability: The cloud provides powerful computing support and cross-regional data integration for agricultural big data; Edge nodes enable real-time local processing and control, enhancing responsiveness and reducing reliance on network connectivity; End devices perform on-site data collection and preliminary processing, supplying essential inputs for higher-level decision-making.

## 4 Typical applications of cloud-edge-device collaboration in smart agriculture

In various subsystems of smart agriculture, cloud-edge-device collaboration has been widely applied due to its distributed architecture, low-latency responsiveness, and multi-layered computing capabilities. This paradigm has proven especially effective in scenarios such as environmental monitoring, irrigation control, agricultural machinery operations, and livestock health management. The following sections present five representative application domains, highlighting typical use cases and their corresponding technical pathways.

### 4.1 Farmland environmental monitoring and data analysis

In smart agriculture systems, farmland environmental monitoring plays a critical role in enabling precision farming, disaster early warning, and resource optimization (Liang et al., 2016; Shi et al., 2018; Chen, 2020; Li et al., 2021; Mahrous Korany Mohamed, 2022; Memon et al., 2023). With the integration of next-generation information technologies such as the Internet of Things (IoT), edge computing, and cloud computing, the cloud–edge–device collaborative architecture has emerged as a foundational technical pathway for the intelligentization of environmental sensing systems (Alharbi and Aldossary, 2021; Saha et al., 2021).

1. Technical architecture and system composition

Following the cloud–edge–device framework (see Section 1), environmental monitoring systems deploy sensors at the device layer, edge nodes for local fusion and alerting, and cloud platforms for trend modeling and early warning. Swain et al. (2023) have shown that the integration of sensor networks with edge nodes in agricultural environments significantly enhances the real-time processing of agricultural data while reducing the communication burden on cloud platforms (Swain et al., 2023; Sutariya et al., 2025). In addition, Zhang et al. (2024) emphasize the importance of data standardization and interoperability across platforms in designing environmental monitoring systems, as these factors are critical for enabling the migration and sharing of agricultural management models across different regions (Zhang J. et al., 2024).

2. Collaboration mechanism and operational strategy

The edge layer offers fast response capabilities and localized processing, enabling immediate detection and response to environmental anomalies. For instance, when a sudden high-temperature event is detected, the edge node can instantly trigger an alarm mechanism and selectively upload relevant data to the cloud for further analysis. Rajora et al. (2023) confirm that the deep integration of IoT with edge computing provides considerable advantages in addressing emergent climatic events and pest or disease outbreaks, thereby enhancing system robustness and responsiveness (Rajora et al., 2023).

**Figure 5:**
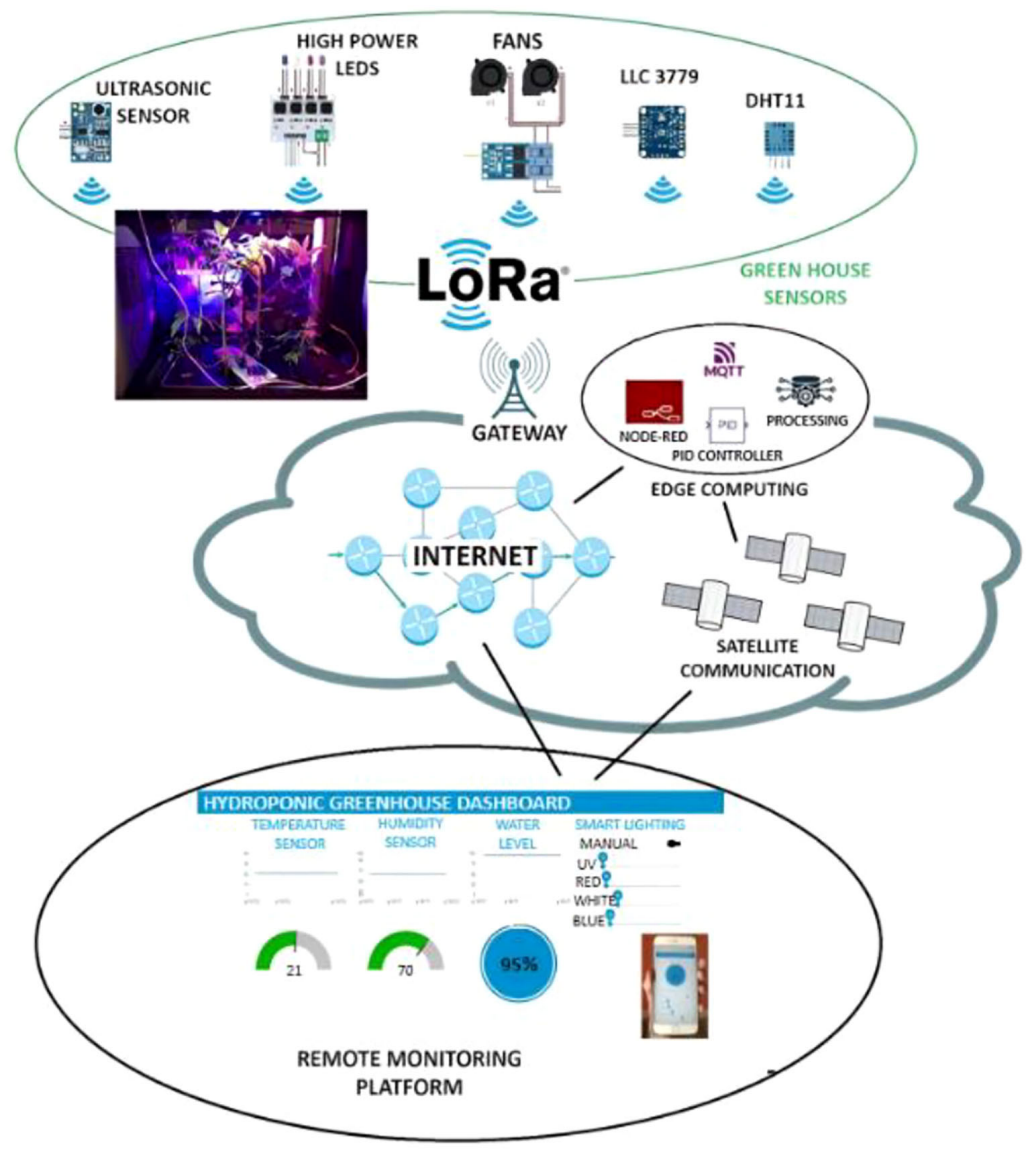
The architecture of an iot-based intelligent hydroponic greenhouse system (Bua et al., 2024).

Meanwhile, the cloud system leverages machine learning and data mining techniques to conduct trend analysis and pattern recognition based on historical datasets, providing data-driven insights for future agricultural decision-making. For example, Bua et al. (2024) proposed the GymHydro system, which combines edge-level control with cloud-based analytics to achieve dynamic regulation of greenhouse environments, significantly improving resource efficiency and environmental stability (Bua et al., 2024). **Figure 5** illustrates the architectural design for transforming a conventional hydroponic greenhouse into an intelligent system. By integrating various IoT technologies, this architecture enables the rapid creation of new services while ensuring key characteristics such as robustness, low power consumption, long-range communication, and reliability. This design provides essential technical support for the intelligent transformation of traditional greenhouses.

3. Application cases and system effectiveness

In recent years, the widespread adoption of cloud–edge–device collaborative architectures in agricultural environmental monitoring has significantly improved the system’s performance in terms of real-time responsiveness, energy efficiency, and scalability. Both academic research and field applications have demonstrated that layered processing strategies enable smart agriculture systems to more effectively sense, analyze, and respond to dynamic environmental changes.

For example, Makondo et al. (2024) proposed a 5G-based edge computing architecture for real-time environmental monitoring in agriculture. By deploying the User Plane Function (UPF) at the network edge and integrating an SDN controller for dynamic data routing, their system achieved over 60% reduction in latency and improved data throughput by approximately 40%, making it highly suitable for time-sensitive tasks such as pest detection and precision irrigation (Makondo et al., 2024).

Similarly, Fatouros et al. (2023) introduced an edge–cloud collaborative architecture built on low-code platforms and functional pattern blocks, designed for real-time monitoring and control in greenhouse agriculture. This system utilized edge devices for local data processing and employed modular development tools to simplify deployment complexity, thereby enhancing system usability and flexibility for smallholder farmers (Fatouros et al., 2023).

Alharbi and Aldossary (2021) developed an energy-optimized hybrid Edge–Fog–Cloud architecture, incorporating a Mixed-Integer Linear Programming (MILP) model to manage real-time data processing and task offloading in agricultural environments. Their experimental results demonstrated substantial improvements, with 36% reduction in total energy consumption, 43% reduction in carbon emissions, and 86% decrease in network congestion when deployed across thousands of sensor nodes—highlighting the value of edge intelligence in large-scale farmland monitoring (Alharbi and Aldossary, 2021).

In a more deployment-oriented study, Garma and Cruz (2024) designed a wireless sensor network (WSN)-based smart irrigation control system that integrates LoRa communication with local edge gateways. The system performs real-time acquisition and analysis of soil moisture, temperature, humidity, and NPK concentration, while enabling autonomous irrigation scheduling. Its architecture minimizes reliance on continuous cloud connectivity, making it ideal for remote agricultural settings (Garma and Cruz, 2024).

**Figure 6:**
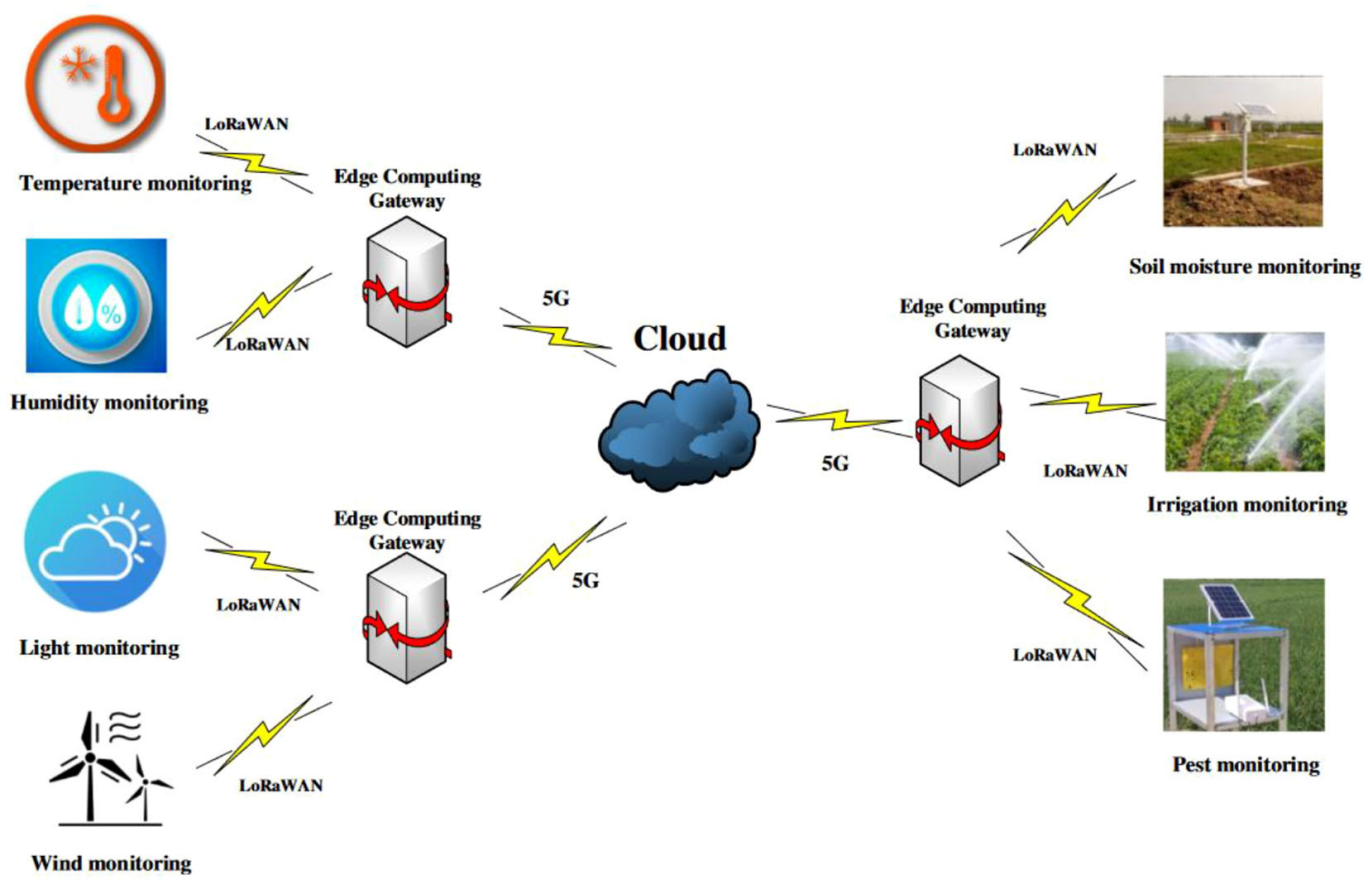
Schematic diagram of agricultural environmental monitoring iot network topology (Dong et al., 2024).

Furthermore, Dong et al. (2024) proposed an edge computing-based farmland monitoring system equipped with deep learning capabilities for pest and disease identification. By deploying a TensorFlow-based image recognition model over a LoRaWAN network, their system achieved a high transmission stability and up to 89% recognition accuracy, significantly reducing the data transmission load traditionally imposed on cloud servers (Dong et al., 2024). **Figure 6** illustrates the topology of the designed IoT network for agricultural environment monitoring. The diagram clearly depicts the connections between the nodes and the gateways within the network, as well as the crucial role of LoRa technology in enabling long-range and efficient data transmission. Through this network architecture, real-time monitoring and data collection of the agricultural environment can be effectively achieved, providing essential technical support for precision agriculture.

4. Summary

Collectively, these studies confirm that the cloud–edge–device architecture exhibits strong adaptability and scalability across diverse agricultural applications. It not only meets the stringent real-time data processing requirements but also supports energy-efficient system operation. As such, it represents a critical enabling framework for building the next generation of intelligent sensing and decision-making systems in smart agriculture.

### 4.2 Intelligent irrigation and greenhouse management systems

(1) Technical architecture and system composition

Intelligent irrigation and greenhouse management systems typically adopt a three-layer collaborative architecture. This hierarchical structure enables dynamic irrigation control and real-time environmental response (Dayioglu, 2014; Preite and Vignali, 2024).

At the perception layer, a range of sensing devices—including soil moisture sensors, ambient temperature and humidity sensors, and weather stations—continuously collect environmental parameters. The edge control layer consists of real-time processing units, often embedded boards based on ARM architecture, which directly drive electromagnetic valves, irrigation pumps, ventilation systems, humidifiers, and shading mechanisms. The cloud platform is responsible for data analytics, crop growth modeling, and decision-making support. By integrating weather forecasts and crop-specific evapotranspiration models, the cloud can generate irrigation optimization strategies for the next 24 hours or even across the entire growing season and issue operational instructions to edge nodes accordingly (Méndez-Guzmán et al., 2022; Benyezza et al., 2023; Arreaga et al., 2024).

This system emphasizes multi-level intelligence. On the edge side, when soil moisture drops below a preset threshold, an immediate irrigation action can be triggered to ensure low-latency local response. Meanwhile, the cloud layer develops mid- to long-term control strategies based on extended weather predictions and crop growth phases, allowing for macro-level resource scheduling and precise regulation of irrigation frequency, ventilation, and shading (Lakhiar et al., 2024).

(2) Application cases and system effectiveness

The three-tier smart irrigation system developed by Et-taibi et al. (2024) exemplifies this architecture. It employs fuzzy logic controllers at the edge for on-site decision-making, while the cloud platform handles data analytics and strategic evaluation. This system demonstrated significant benefits in both water conservation and crop health within a soilless drip irrigation context (Et-taibi et al., 2024).

**Figure 7:**
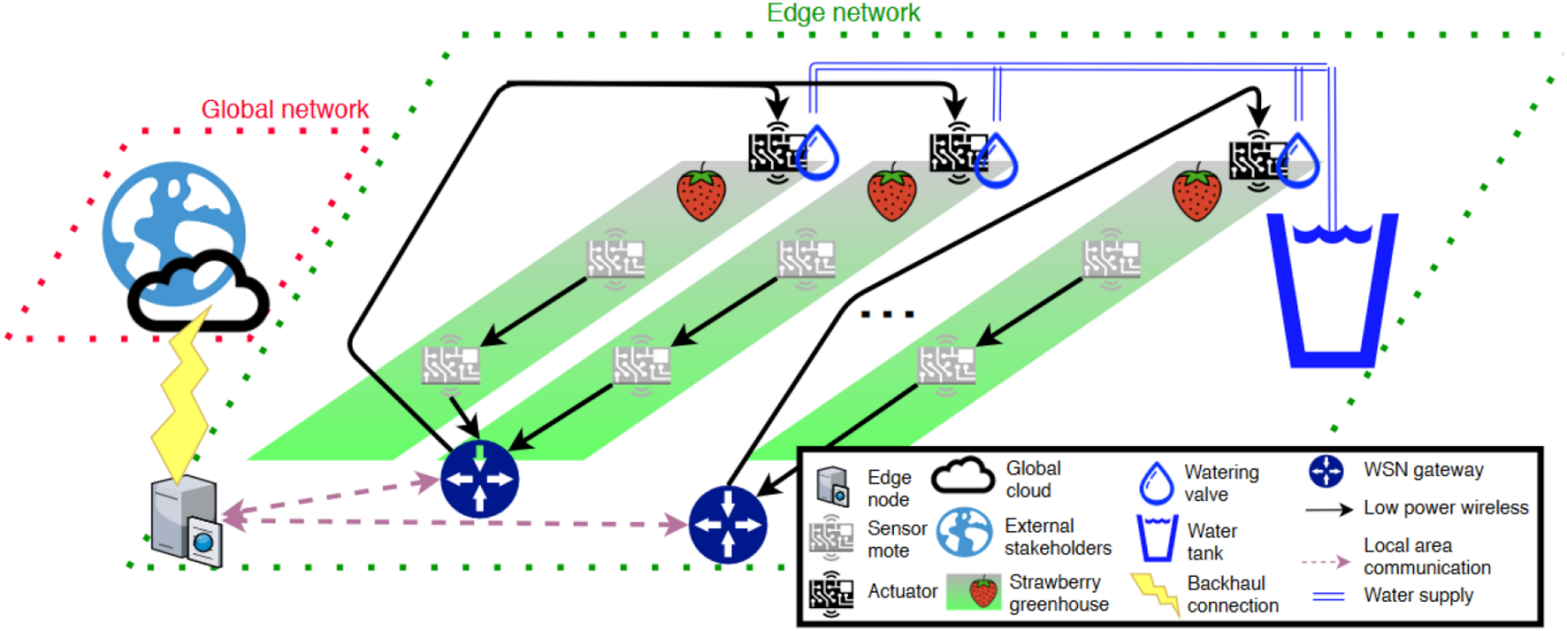
Proposed architectural design for SINs applied to strawberry greenhouses (Angelopoulos et al., 2020).

In another study, Angelopoulos et al. (2020) introduced a highly localized edge irrigation system in a strawberry greenhouse, minimizing dependence on cloud connectivity. This design improved data security, response time, and adaptability, and significantly mitigated soil moisture variability while optimizing water use efficiency (Angelopoulos et al., 2020). **Figure 7** provides a detailed illustration of how the design optimizes the intelligent management of strawberry greenhouses through edge computing and localized data processing, while ensuring data security and privacy.

Additionally, Fatouros et al. (2023) leveraged low-code development tools to rapidly construct an edge–cloud collaborative greenhouse control system. Through graphical functional blocks, the system allows precise edge-side control over irrigation, ventilation, and humidification operations. Meanwhile, cloud-side modules support strategy iteration and decision optimization, reducing deployment complexity and improving scalability (Fatouros et al., 2023).

In the context of IoT-based smart greenhouse optimization, Ariss et al. (2024) developed a zonal control system for tomato cultivation. Their architecture integrated edge computing, 5G communication, and automated sensory feedback, enabling differentiated irrigation and environment regulation for various growth zones, which led to substantial improvements in crop yield and quality (Ariss et al., 2024).

Raro and Palaoag (2024), on the other hand, focused on human–machine interaction. They developed a mobile platform for real-time environmental monitoring and remote irrigation management, which proved effective in diverse climatic regions and suitable for broader agricultural deployments (Raro and Palaoag, 2024).

(3) Summary

Overall, intelligent irrigation and greenhouse management systems based on the sensor–edge controller–cloud platform collaborative architecture exhibit significant advantages in real-time responsiveness, strategy optimization, and environmental adaptability. By integrating local edge control with cloud-based decision support, these systems enable fine-grained management of water and nutrient resources, enhance crop yield and quality, and drive the green and intelligent transformation of modern agriculture.

### 4.3 Collaborative operations of agricultural uavs and ground machinery

(1) Technical architecture and system composition

In smart agriculture, the collaborative operation of unmanned aerial vehicles (UAVs) and agricultural machinery is progressively evolving into a three-tier intelligent system based on end–edge–cloud collaboration. This architecture typically consists of multiple UAVs (equipped with multispectral cameras and spraying modules), autonomous navigation machinery (e.g., smart tractors), RTK-based high-precision positioning modules, edge scheduling platforms, and cloud-based operational systems (Popescu et al., 2020; Ren et al., 2024).

Edge nodes utilize wireless communication technologies such as 5G, LoRa, or WiFi Mesh to establish real-time communication and spatial awareness between UAVs and ground equipment. Meanwhile, the cloud platform undertakes core functions including task management, path planning, data archiving, and operation optimization.

(2) Coordination mechanism and operational logic

The collaborative mechanism is designed with division of labor across layers. The edge platform collects and synchronizes real-time operational states and spatial positions of all field devices. It performs low-latency task scheduling, obstacle detection, and dynamic collision avoidance, ensuring operational safety and efficiency (Kalyani and Collier, 2021; Qu et al., 2022). The cloud layer supports adaptive task allocation based on historical operations and real-time environmental dynamics (e.g., crop health imagery, pest outbreak predictions), dynamically adjusting execution plans as conditions change (Zhu, 2018; Xu et al., 2019; Chen, 2020; Ahmed et al., 2021; Lian, 2021; Zhu et al., 2022; Cong et al., 2023; Wei et al., 2023; Xu S. et al., 2023; Feng et al., 2024; Gao et al., 2024; Zhang L. et al., 2024).

Coordination among multiple machines is typically enabled by distributed control algorithms or leader–follower strategies, which ensure the stable and scalable deployment of dense UAV-ground systems across large farmland areas.

(3) Application cases and system effectiveness

In recent years, collaborative operations of agricultural unmanned systems (e.g., UAVs and unmanned ground vehicles, UGVs) have emerged as a critical focus in smart agriculture. Extensive research has explored key challenges including operation optimization, path planning, and task scheduling under the end–edge–cloud framework, resulting in a range of innovative architectures and solutions.

Lee et al. (2021) proposed a multi-UAV coordinated system employing a leader–follower formation strategy and real-time control using the Robot Operating System (ROS). The system enabled synchronized image acquisition from multiple perspectives, enhancing phenotypic reconstruction accuracy and measurement efficiency. Their findings also showed that UAV cooperation reduces error and improves task efficiency under wind disturbances, offering a robust solution for agricultural remote sensing (Lee et al., 2021).

Qu et al. (2022) provided a comprehensive overview of UAV swarm applications in smart agriculture. They emphasized the advantages of UAV clusters in crop diagnosis, pesticide spraying, and map generation over single-drone operations, particularly in terms of parallel task execution and response speed. Their review also highlighted the importance of resource scheduling and edge computing support, particularly for task segmentation, energy management, and interference mitigation (Qu et al., 2022).

Uddin et al. (2021) introduced the concept of Flying Edge Computing, where UAVs function as temporary edge nodes to conduct high-frequency data collection and localized analysis. This architecture is particularly suited to challenging terrains such as hilly or remote farmlands, where fixed edge infrastructure may be difficult to deploy or maintain (Uddin et al., 2021). The proposed system consists of two primary components: an edge device and the Flying Edge computing machine. The edge devices (such as sensors or actuators) request and receive services directly from a UAV through a dedicated service request interface. Most IoT computational tasks are handled locally by the UAV’s service response module, which hosts various integrated services. Occasionally, when IoT devices require more extensive or global resources, these services are accessed through a global service response module connected to cloud resources (Uddin et al., 2021).

Shi et al. (2023) addressed air–ground collaborative path planning, proposing a dynamic path optimization algorithm that combines K-means clustering with Markov chain modeling. UAVs assist in obstacle detection and relay information to guide ground machinery, using historical data for predictive state transitions that significantly improve operational efficiency and safety (Shi et al., 2023).

**Figure 8:**
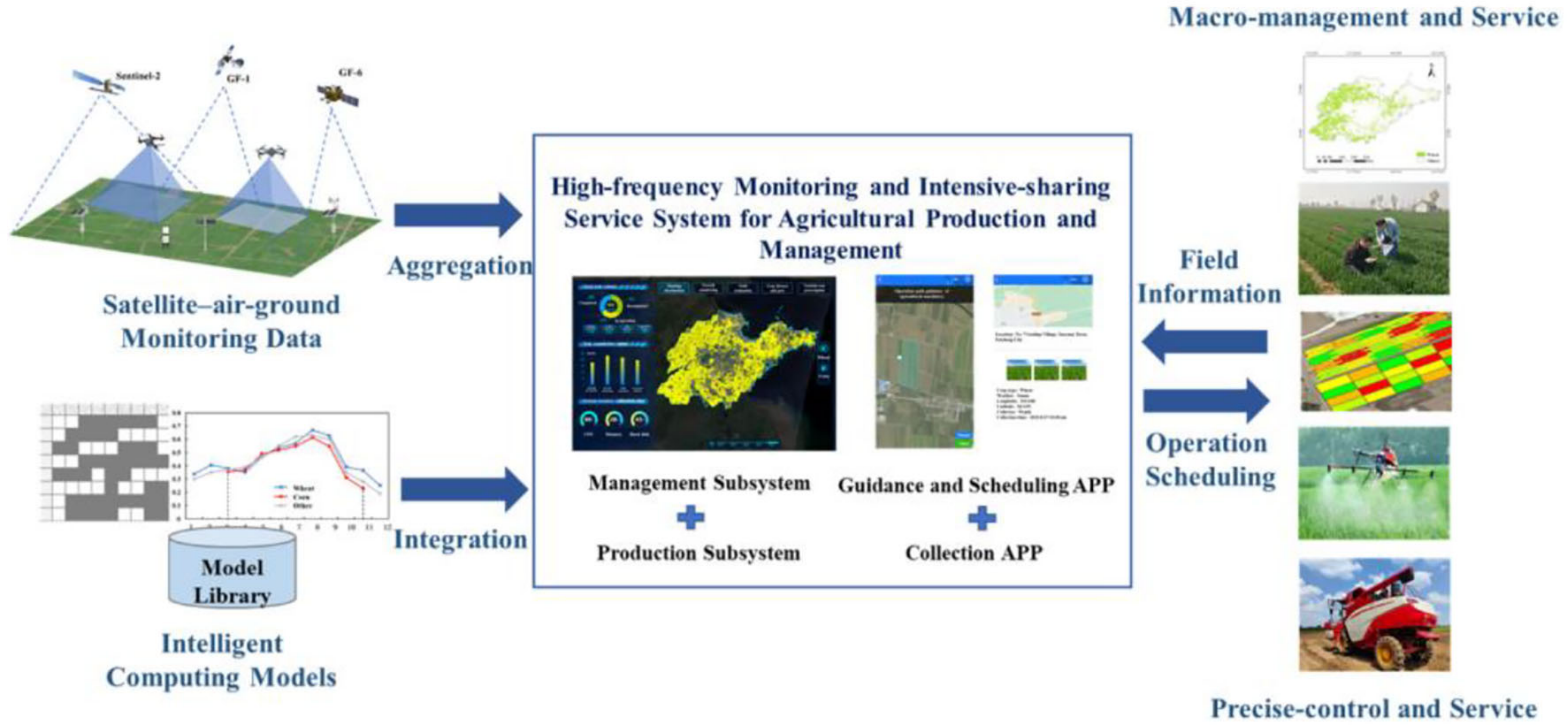
The workflow of the high-frequency monitoring and intensive sharing service (Li W. et al., 2023).

Li et al. (2023) explored an air–ground–satellite integrated sensing system, showcasing the synergistic use of UAVs and ground machinery for macro-level crop monitoring and regional task coordination. A pilot deployment in Shandong Province demonstrated the system’s ability to perform high-frequency agricultural monitoring, supporting both government oversight and fine-grained field operations through layered IoT sensing networks (Li W. et al., 2023). **Figure 8** illustrates the workflow of the high-frequency monitoring and intensive sharing service (Li W. et al., 2023).

(4) Summary

Collaborative operations between UAVs and agricultural machinery via end–edge–cloud architecture are becoming foundational to smart agriculture. By combining real-time situational awareness and control at the edge with strategic planning at the cloud, these systems enhance both operational efficiency and field safety. Future research should prioritize the development of multi-agent collaboration strategies, resilient network designs, and cross-regional task optimization models to meet the increasing diversity and complexity of agricultural operations.

### 4.4 Livestock behavior recognition and health monitoring

(1) Technical architecture and system composition

In smart livestock farming, the cloud–edge–device framework (Section 1) unifies real-time behavior recognition and health monitoring (Park and Park, 2020; Shen et al., 2021). Wearable tags stream vital-sign and activity data to on-premise edge boxes (Jetson Nano, etc.) that run lightweight models for immediate filtering, behavior classification, and anomaly alerts. The cloud receives only condensed, high-value events and longitudinal data, enabling herd-level trend modelling, disease-outbreak prediction, and risk-driven management recommendations (Huang et al., 2018; Chen et al., 2020; Pan et al., 2023).

(2) Coordination Mechanism and Operational Logic

In a typical operational scenario, edge devices are capable of identifying aggressive interactions, falls, and abnormal locomotion in livestock locally and issuing alerts within milliseconds. Meanwhile, the cloud platform aggregates historical health data from different herds and regions and applies deep learning or expert-rule models to forecast chronic conditions and epidemic risks. Some systems also incorporate modules for automated feeding, environmental control, and emergency response, enabling a closed-loop workflow from “perception” to “cognition” to “reaction.”

(3) Application cases and system effectiveness

With the integration of artificial intelligence, edge computing, and the Internet of Things (IoT), livestock health monitoring is evolving toward intelligent, real-time, and cooperative models. Recent research efforts have established a technical foundation for cloud–edge–device architecture in the livestock domain, focusing on behavior recognition algorithms, edge AI frameworks, and cross-cycle health modeling.

In Terms of System Integration and Intelligent Perception. Arora et al. (2024) (Harish Arora et al., 2024) proposed the “Smart Cattle Care” system architecture, which integrates IoT terminals (for temperature, heart rate, and positioning), edge inference modules, and a cloud-based health management platform. Their study emphasizes the importance of multimodal sensor fusion and redundant data cleansing in improving diagnostic model accuracy. Behneghar et al. (2021) developed a cattle behavior classification system based on KNN and CNN algorithms. Using LoRa for energy-efficient data transmission between sensors and edge platforms, they demonstrated that while CNN achieved higher accuracy (~84%), KNN offered better real-time performance and portability for resource-constrained devices (Behneghar et al., 2021). Nikam et al. (2019) (Nikam et al., 2019)explored the feasibility of low-cost perception systems for small-scale farms, demonstrating the potential of simplified sensing and basic edge logic as a viable model for smart livestock farming in developing regions and remote areas.

**Figure 9:**
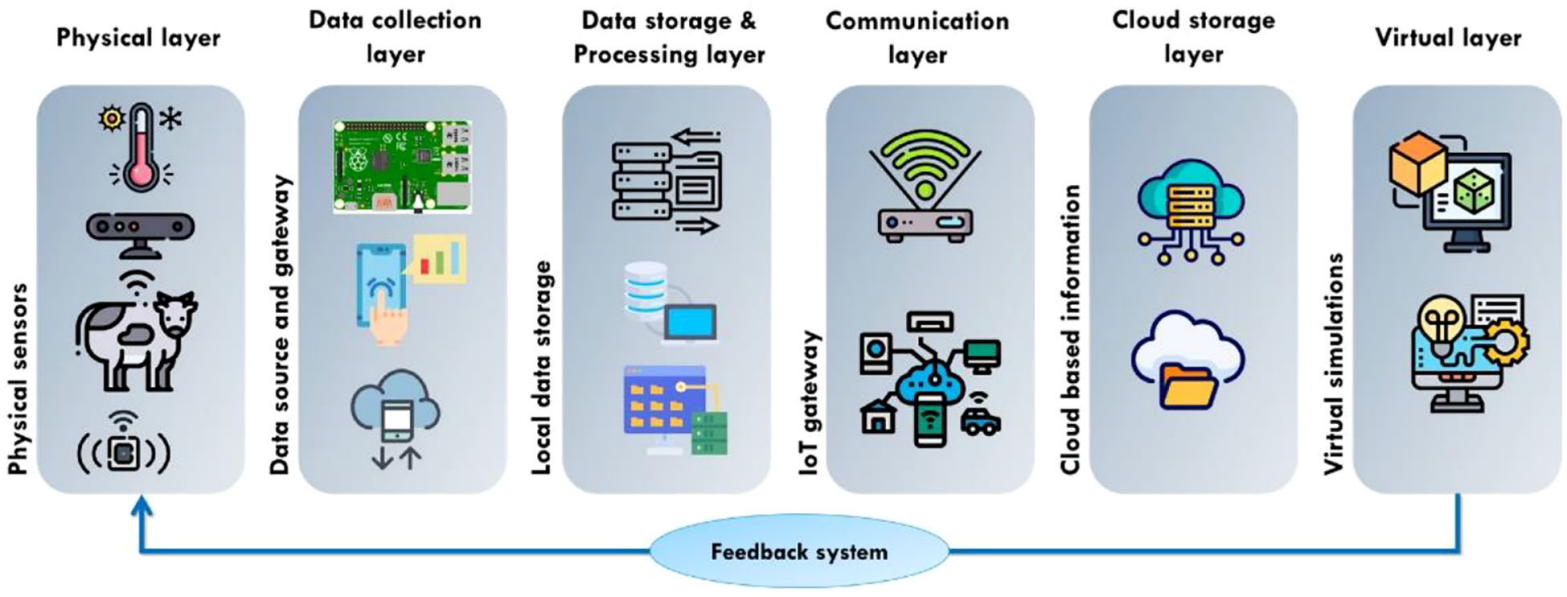
Digital twin with six layered architectures (Arulmozhi et al., 2024).

In Terms of Edge Intelligence and Real-Time Recognition. Öztürk et al. (2020) developed the “Livestock Welfare Platform,” a hybrid edge–blockchain system enabling localized data processing and provenance tracking. The platform delivers high responsiveness in monitoring animal movement, feeding behavior, and estrus cycles, and records critical events on the blockchain for transparency and traceability (Zervas et al.,, 2021). Park and Park (2020) proposed a behavior trend analysis system combining cloud platforms with sensor networks. Using centralized time-series clustering, the system detects latent activity patterns to compute individual health scores and group behavior indices, serving as a representative edge-judgment–cloud-modeling approach (Mao et al., 2023). Arulmozhi et al. (2024) introduced a digital twin–based virtual livestock farm management system. By synchronizing “virtual cattle” models with real-time edge data, the platform enables remote anomaly detection and intervention suggestions. Its high scalability and cross-platform compatibility support deployment in diverse livestock scenarios (Arulmozhi et al., 2024). As shown in **Figure 9**, the proposed six-layer digital twin architecture provides a systematic solution for integrating physical devices with virtual models. Future research could further focus on the application and optimization of this architecture in specific industrial scenarios.

**Figure 10:**
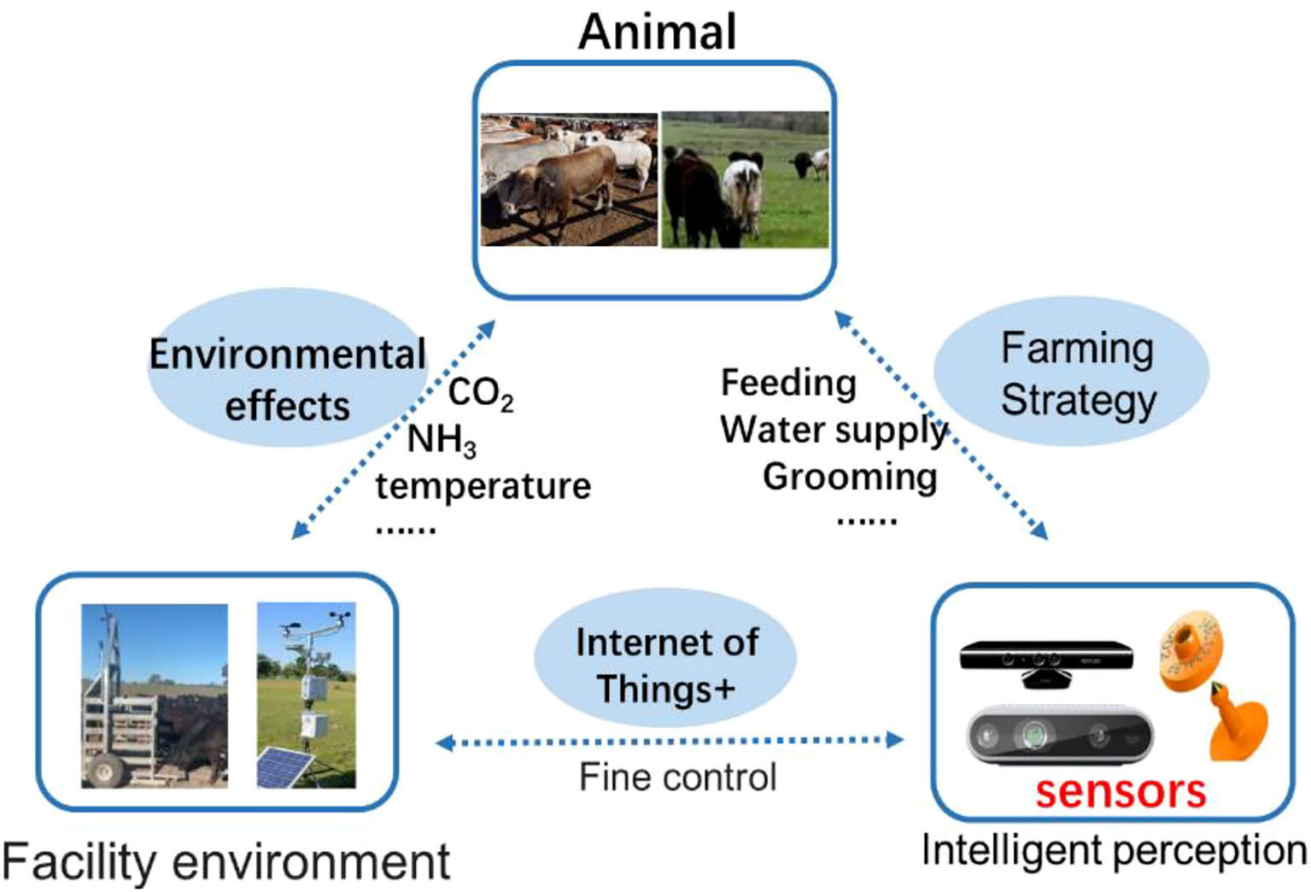
The framework of intelligent perception-based animal farming.

In Terms of Theoretical Perspectives and Future Directions. Qiao et al. (2021) identified several key issues in current livestock behavior recognition systems, including “data silos,” weak model generalization, and insufficient multimodal data fusion. They recommend future work focus on sensor data calibration, model compression for edge deployment, and enhanced animal identification algorithms—supported by multitask learning strategies to improve accuracy and generalizability (Qiao et al., 2021). **Figure 10** illustrates the proposed general intelligent perception-based animal farming process. Similarly, Li et al. (2023) emphasized the emerging trend of multimodal systems that integrate machine vision, video analytics, audio detection, and physiological sensing for large-scale ranches. However, such systems demand robust high-bandwidth connections and efficient edge processing algorithms to operate effectively (Li W. et al., 2023).

(4) Summary

The cloud–edge–device collaborative architecture is driving a paradigm shift in livestock health monitoring, transitioning from reactive interventions to proactive recognition and predictive warning. Future research should continue to explore multi-source heterogeneous data integration, lightweight edge AI algorithms, and the joint optimization of data privacy and model interpretability to support more intelligent, efficient, and sustainable livestock production systems.

### 4.5 Pest and disease monitoring and early warning

(1) Technical architecture and system components

In modern smart agriculture, pest and disease intelligent monitoring and precise early warning leverage the cloud-edge-device architecture (outlined in Section 1) for distributed sensing and coordinated response. Specifically, lightweight CNN or YOLO models hosted on edge nodes perform real-time image screening and anomaly detection in the field; only high-risk samples are uploaded to the cloud (Cardoso et al., 2022; Kum et al., 2024). The cloud platform then fuses historical disease data, weather information, and crop growth cycles to train spatiotemporal prediction models and generate regional control recommendations, forming a closed loop of “edge filtering–cloud analysis–local response” (Yang et al., 2019; Jin, 2021).

(2) Coordination mechanism and response strategy

During operation, edge devices perform tasks such as pest image capture, regional object detection, and primary classification. When abnormal pest activity is detected—e.g., insect density exceeding a threshold—relevant feature images and structured data are transmitted to the cloud. The cloud platform then integrates historical pest distribution, weather dynamics, and crop growth cycles to construct spatiotemporal prediction models. These models generate personalized pest management recommendations, which are sent back to local agricultural control systems, forming a closed-loop workflow of “detection–analysis–response.”

(3) Application cases and system effectiveness

Recent years have seen increasing attention to the application of cloud–edge–device frameworks in pest and disease control. Zhang et al. (2021) proposed a Cooperative Edge–Gateway Computing Architecture (CGET), which implements a two-layer structure comprising smart edge gateways and edge servers. This setup enables localized image compression and filtering, preserves data privacy, and improves responsiveness and task offloading efficiency (Zhang et al., 2021).

Vo et al. (2022), in a comprehensive review of edge–cloud collaborative architectures, highlighted that pest monitoring is a low-latency, high-density scenario. System efficiency heavily depends on edge-level preprocessing and object compression strategies. Sole reliance on cloud computing, they argued, can lead to data bottlenecks and delayed response, ultimately compromising the timeliness of warnings.

Wang et al. (2022) introduced a sliding-mode control mechanism combined with network virtualization to ensure quality-of-service (QoS) in agricultural video monitoring and pest detection. Their approach significantly enhances the dynamic resource allocation and network stability of edge nodes and provides valuable insight for event-driven pest identification systems in precision agriculture (Vo et al., 2022).

Ma (2024) proposed a cloud–edge collaborative data stream processing model tailored for IoT-based smart agriculture. When applied to pest recognition systems, the model reduced data transmission latency by nearly 40% and improved response speed by over 30%, providing essential hardware and scheduling support for the deployment of complex image recognition algorithms at the edge (Wang et al., 2022).

(4) Summary

In summary, pest and disease monitoring systems built upon the coordinated flow of “sensor terminal–edge recognition–cloud prediction” have greatly improved detection accuracy and system responsiveness. These systems also mark a paradigm shift from experience-based to data-driven pest management. Future research should focus on: lightweight compression of pest recognition models for edge deployment; multimodal data fusion integrating vision, meteorological, and historical pest information; cross-regional model transfer and generalization; and integration with intelligent spraying systems to establish an end-to-end prevention–response chain.

### 4.6 Comparison of cloud–edge–device application scenarios

To comprehensively understand the technical implementation and application effectiveness of cloud–edge–device collaborative architectures in smart agriculture, a comparative analysis of typical application scenarios is presented in **Table 1**. This table summarizes the roles and interactions of terminal, edge, and cloud layers across five representative domains: environmental monitoring, intelligent irrigation, UAV and machinery coordination, livestock health monitoring, and pest/disease early warning. By contrasting their functional distributions and system advantages, this analysis highlights the architectural flexibility and scenario-specific optimization potential of cloud–edge–device collaboration. The comparison also provides insight into how different system layers contribute to achieving real-time response, precision control, and efficient resource utilization under diverse agricultural conditions.

**Table 1:** Comparative table of typical application scenarios for cloud–edge–device collaboration.

Application scenario	Terminal layer function	Edge layer processing	Cloud functionality	Advantages
Farmland Environmental Monitoring	Real-time data collection via multi-type sensors	Data fusion and local alerting	Historical data trend analysis	Faster response and reduced data redundancy
Intelligent Irrigation & Greenhouse Control	Perception via humidity/temperature sensors	Local threshold decision-making and control	Crop modeling and weather forecasting	Precision control, water and energy saving
UAV & Agricultural Machinery Coordination	Sensing and navigation by drones and tractors	Task scheduling and obstacle avoidance	Multi-day task planning and workload distribution	Efficient operations and coordinated scheduling
Livestock Behavior & Health Monitoring	Wearable devices and video data collection	Behavior recognition and health warnings	Health modeling and diagnostic support	Early warnings, loss reduction
Pest and Disease Monitoring and Early Warning	Image capture and insect density sensing via cameras	On-edge detection (e.g., YOLO), threshold-based screening	Spatiotemporal prediction and decision support	Timely warnings, localized control, reduced bandwidth load

## 5 Discussion

The widespread deployment of cloud–edge–device collaborative computing in smart agriculture has demonstrated significant potential in enhancing system responsiveness, reducing bandwidth pressure, and enabling distributed intelligence. However, despite the promising progress in specific application domains such as irrigation, pest detection, and livestock monitoring, several critical challenges persist that hinder large-scale, robust, and interoperable implementations. This section synthesizes the findings from the reviewed literature, identifies key limitations, and outlines urgent research directions.

### 5.1 System integration vs. modular fragmentation

While modular solutions (e.g., lightweight edge AI models, LoRa-based sensing, UAV-UGV coordination strategies) have shown strong performance in isolated tasks, the lack of end-to-end system integration remains a major barrier. Most studies focus on optimizing individual components without validating their interoperability or performance in real-world, multi-tasking environments. This fragmentation leads to duplicated efforts, incompatible protocols, and difficulty in scaling up. Future research must prioritize system-level design frameworks that support seamless integration across heterogeneous devices, platforms, and communication standards.

### 5.2 Data inconsistency and cross-platform interoperability

Agricultural environments are inherently heterogeneous, involving diverse sensor types, data formats, and communication protocols. The absence of standardized data models and semantic interoperability across edge and cloud layers limits the generalizability of AI models and decision-support systems. For instance, a pest detection model trained in one region may fail in another due to differences in image resolution, lighting, or pest species. Developing ontology-driven data fusion frameworks and open agricultural data standards is essential to ensure cross-regional model transferability and system interoperability.

### 5.3 Real-time performance vs. resource constraints

Although edge computing significantly reduces latency, it also introduces trade-offs between real-time responsiveness and resource efficiency. Many edge devices are constrained by limited memory, processing power, and energy supply. Current literature often reports algorithmic accuracy without addressing energy consumption, hardware cost, or deployment feasibility. To bridge this gap, future studies should incorporate co-design principles that jointly optimize algorithm efficiency and hardware constraints, and provide benchmark datasets for fair comparison across platforms.

### 5.4 Privacy, security, and governance

Agricultural data often contains sensitive information, such as farm location, crop yield, and management practices. Centralized data collection poses significant privacy risks. While federated learning and edge-based processing offer partial solutions, they introduce new challenges in data governance, model accountability, and secure aggregation. Moreover, most existing works overlook socio-technical aspects, such as farmer trust, regulatory compliance, and economic incentives. A multi-disciplinary approach—combining technical innovation with policy, ethics, and usability studies—is urgently needed.

### 5.5 Reproducibility and benchmarking deficits

A notable limitation in the current literature is the lack of reproducible experiments and open benchmarks. Many studies are based on proprietary datasets or small-scale simulations, making it difficult to validate or compare results. This undermines scientific rigor and slows down collective progress. Establishing open-access agricultural datasets, standard evaluation metrics, and reproducible experimental protocols is critical for advancing the field.

### 5.6 Toward scalable and resilient systems

Ultimately, the transition from proof-of-concept to practical deployment requires resilient, scalable, and maintainable systems. This includes not only robust algorithms and architectures but also lifecycle management, remote update mechanisms, and fault-tolerant designs. Future research should explore digital twins and simulation environments for pre-deployment testing, as well as human-in-the-loop frameworks to enhance system adaptability and trust.

### 5.7 Research directions ahead

To address the above challenges, we identify the following priority research directions:

1. Integrated system architectures that unify cloud, edge, and device layers under open standards;
2. Energy-aware and cost-effective edge AI models tailored for agricultural scenarios;
3. Privacy-preserving federated learning frameworks with real-world validation;
4. Ontology-based semantic fusion for cross-platform interoperability;
5. Open benchmarks and reproducibility protocols for fair evaluation;
6. Socio-technical studies on usability, trust, and adoption barriers.

## 6 Future trends and research prospects

As smart agriculture enters a more advanced stage of development, cloud–edge–device collaborative architectures for sensing, analysis, and control are evolving from static connectivity models toward intelligent, adaptive, and distributed autonomous systems. Looking ahead, several challenges remain in terms of system intelligence, scalability, data privacy, and cross-domain operability. To address these challenges, future research should focus on the following six key directions:

### 6.1 Intelligent collaborative scheduling algorithms for agricultural cloud–edge–device systems

As smart agriculture systems become increasingly complex, the challenges posed by terminal heterogeneity, task density, and dynamic environments demand highly responsive and adaptive resource scheduling capabilities. Within the three-tier architecture of cloud–edge–device collaboration, it is essential to dynamically perceive device status, network topology, and agricultural task priorities in real time to develop flexible, intelligent, and robust scheduling mechanisms. Traditional static allocation strategies fall short in handling frequent emergencies and fluctuating resource availability in agricultural settings, thus calling for the adoption of adaptive intelligent scheduling algorithms.

In recent years, multi-agent reinforcement learning (MARL) has emerged as a promising approach for solving complex scheduling problems. For instance, Gu et al. (2024) proposed an AI-enhanced collaborative scheduling network that utilizes deep reinforcement learning to formulate cross-layer task offloading strategies. Their system effectively reduces average latency and improves load balancing and scheduling elasticity under multi-resource constraints (Gu H. et al., 2024). Similarly, Liang et al. (2024) integrated digital twin technology to construct a cross-regional edge node coordination network that enables device state mapping and policy prediction, thereby optimizing collaborative scheduling strategies and system-level visualization (Liang et al., 2024).

Xu et al. (2023) introduced virtual object management into the scheduling process by enabling edge devices to track the status of virtual farm entities. This approach enhances context-aware scheduling and allows timely responses to agricultural changes, significantly improving the adaptability of scheduling systems (Xu R. et al., 2023). In addition, Zhang et al. (2022) proposed a deadline-aware edge–cloud scheduling mechanism in the industrial IoT domain, offering valuable insights for addressing hard real-time execution requirements (Zhang et al., 2022b). Li et al. (2024) further advanced the field by developing a multi-agent attention-based scheduling architecture that considers both task prioritization and energy efficiency, enhancing decision-making and load balancing in agricultural edge scenarios (Li Z. et al., 2024).

Looking forward, research on scheduling algorithms for smart agriculture should explore the following directions:

1. Incorporating graph neural networks (GNNs) to model edge network topologies and spatiotemporal communication patterns, improving global awareness in scheduling decisions;
2. Developing heterogeneous resource modeling and joint scheduling strategies, enabling coordinated evaluation of computing, storage, and communication capacities;
3. Designing transferable and generalizable scheduling models to accommodate diverse environments, crop types, and agricultural cycles;
4. Integrating energy consumption modeling and green computing paradigms to balance computational efficiency and sustainability;
5. Combining digital twin technology with scheduling processes, allowing simulation-based pre-evaluation and optimization of scheduling decisions in virtual environments.

### 6.2 Deep integration of edge ai and lightweight models in smart agriculture

As smart agriculture increasingly relies on real-time sensing and intelligent decision-making, edge computing has emerged as a critical enabling technology. However, edge devices typically suffer from limited computational resources, constrained energy consumption, and harsh operating environments, which present challenges to the stable deployment of traditional large-scale deep learning models. In this context, the core objective of edge AI development is to design lightweight, efficient, and transferable neural network models capable of performing intelligent perception and local inference under resource-constrained conditions (Pei et al., 2022; Zhang et al., 2023; Guan et al., 2024; Li A. et al., 2024; Zuo et al., 2024).

Current research focuses on several model optimization strategies, including pruning, quantization, architectural reorganization (e.g., depthwise separable convolutions), and knowledge distillation. For instance, Kalyani et al. (2023) deployed a pruned and quantized convolutional neural network (CNN) in an intelligent greenhouse system. By fusing thermal imaging and illumination sensor data, their model achieved low-power yet high-accuracy identification of plant growth status (Kalyani et al., 2023).

Praharaj et al. (2023) embedded long short-term memory (LSTM) networks within a federated learning framework to perform localized time-series anomaly detection across agricultural nodes. Their approach enhanced responsiveness to climatic fluctuations and disease outbreaks while reducing communication overhead through lightweight parameter exchanges (Praharaj et al., 2023).

Zeng et al. (2022) proposed a hybrid optimization method combining particle swarm optimization (PSO) and pruning strategies to implement collaborative task scheduling. Their solution significantly reduced model complexity while preserving accuracy, enabling rapid pest image recognition on ARM-based edge devices (Zhang et al., 2023).

Looking forward, several key directions merit further exploration:

1. Neural Architecture Search (NAS) for Edge Deployment: Develop adaptive models tailored for edge environments using NAS, while incorporating hardware constraints for joint optimization.
2. Standardization of Inference Frameworks and Cross-Platform Compatibility: Promote unified use of lightweight inference platforms such as TensorFlow Lite, PyTorch Mobile, and ONNX Runtime in agricultural edge deployments.
3. Self-Compressing and Self-Distilling Models: Design models capable of online compression and self-distillation to improve adaptability and generalization across diverse agricultural scenarios.
4. Scenario-Specific Lightweight Model Libraries: Establish modular libraries of lightweight models for targeted tasks such as rice disease identification, greenhouse regulation, and pest prediction to enhance reusability.
5. Multimodal Edge Inference: Strengthen edge models’ ability to integrate and infer from heterogeneous data sources, including images, audio, and weather information, to support more comprehensive agricultural decision-making.

### 6.3 Constructing multi-level data fusion and hierarchical decision-making frameworks

In agricultural IoT environments, dense sensor networks generate vast volumes of heterogeneous data—including meteorological readings, soil measurements, image streams, video surveillance, and agricultural machinery operation logs. These data exhibit spatial heterogeneity, multi-scale temporal distributions, and often involve redundancy, uncertainty, and complex cross-layer interactions. Therefore, developing an efficient data fusion and hierarchical decision-making architecture is essential for enhancing the intelligence and responsiveness of smart agriculture systems (Yu et al., 2024).

Su et al. (2022) proposed a two-level optimization model based on cloud–edge collaboration. In this framework, edge nodes are responsible for the local processing of short-cycle data (e.g., temperature, humidity, light intensity), while the cloud layer performs trend modeling and regional forecasting based on historical datasets. This design achieves a balance between localized responsiveness and global optimization (Su et al., 2022).

**Figure 11:**
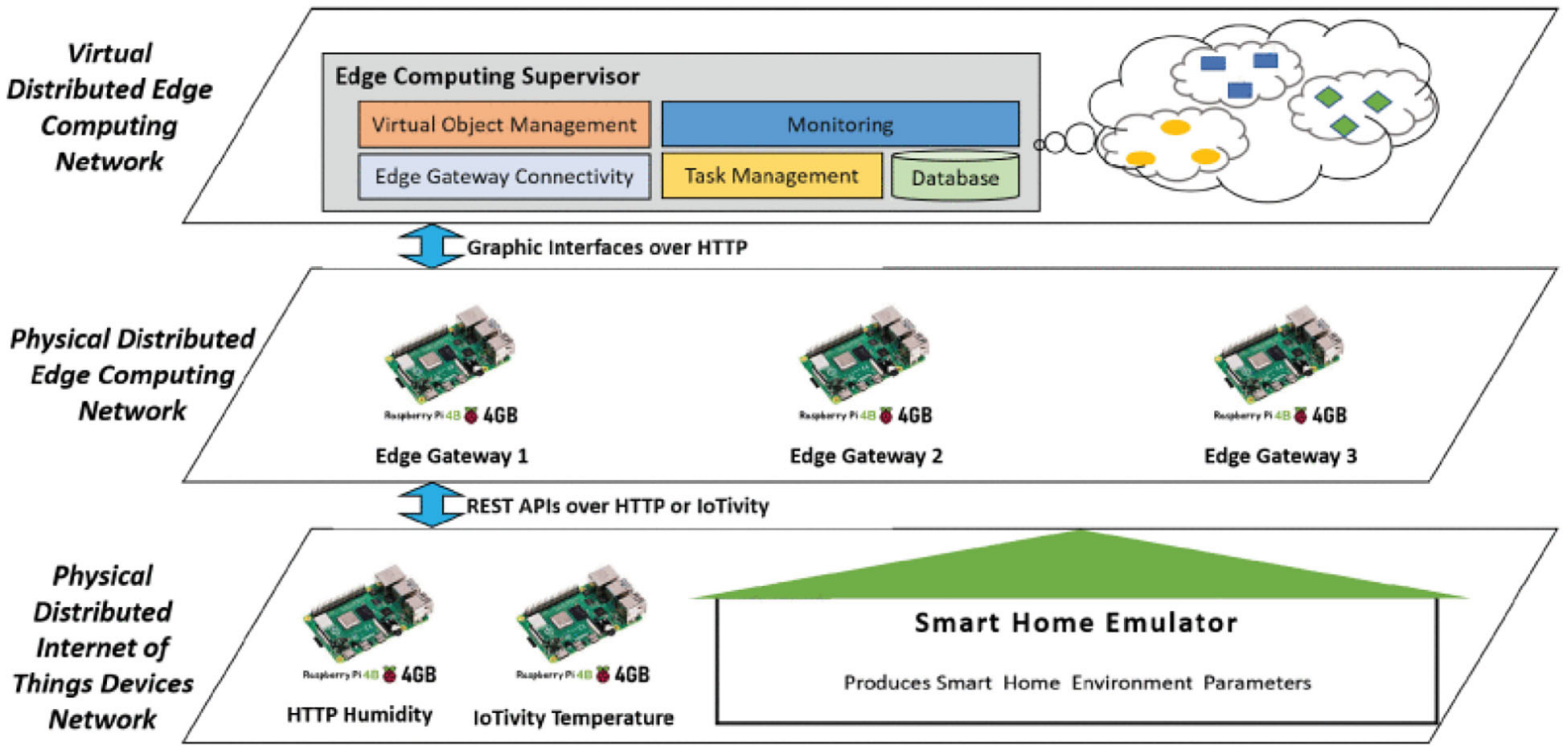
Proposed development architecture for distributed edge computing environment (Xu R. et al., 2023).

Moreover, Xu et al. (2023) introduced a virtual object (VO)-based cloud–edge perception system, allowing each edge device to maintain a local VO replica. This supports synchronized status prediction and facilitates cross-scenario data association and interregional fusion on the cloud platform (Xu R. et al., 2023). **Figure 11** illustrates the proposed development architecture for a distributed edge computing environment.

Future system designs may further evolve along the following directions:

1. Semantic fusion and ontology-based modeling: Develop unified semantic structures across heterogeneous sources using agricultural knowledge graphs and ontologies to enable integrated interpretation of structured and unstructured data.
2. Multi-scale spatiotemporal fusion: Integrate long-term trends (e.g., climate data) with short-term dynamics (e.g., pest outbreak images) to construct fused models across spatial hierarchies and temporal windows.
3. Hierarchical decision chains for edge–cloud collaboration: Assign edge nodes with rule-based logic, anomaly detection, and alerting tasks, while the cloud handles high-complexity inference driven by large-scale models.
4. Data quality-aware fusion mechanisms: Incorporate indicators such as data integrity, confidence level, and latency to dynamically weight sensing data during the fusion process.
5. Distributed fuzzy reasoning and knowledge-enhanced inference: Combine fuzzy logic and expert systems to improve decision robustness under uncertainty, which is common in agricultural settings.

### 6.4 Federated learning: privacy-preserving distributed intelligence in smart agriculture

In smart agriculture, vast amounts of data related to land location, crop types, and management practices are continuously collected. However, centralized model training poses significant risks to data privacy, thereby limiting data sharing and collaborative modeling capabilities. Federated Learning (FL) offers a promising paradigm that enables local model training without uploading raw data. Instead, only model parameters are shared, allowing distributed intelligent modeling under privacy-preserving constraints.

Recent studies have highlighted the potential and applicability of FL in agricultural contexts. For instance, Dembani et al. (2025) emphasized that FL can be applied to various tasks such as pest and disease identification, yield prediction, and resource optimization while ensuring data privacy. Nonetheless, they also pointed out practical challenges, including data heterogeneity, communication latency, and limited computational power (Dembani et al., 2025). In real-world deployments, Dwarampudi and Yogi (2024) proposed an FL-based agricultural intelligence system that enables personalized model training at the farm level while aggregating model parameters from different regions to produce localized predictions (Dwarampudi and Yogi, 2024).

Meanwhile, Singhal (2025) reviewed privacy-preserving mechanisms used in FL, including Differential Privacy (DP), Homomorphic Encryption (HE), and Secure Aggregation protocols. These methods enhance privacy protection but remain constrained by computational overhead and communication complexity (Singhal, 2025).

From a feasibility and performance perspective, Siniosoglou et al. (2023) embedded Long Short-Term Memory (LSTM) models within a federated framework to forecast crop trends across multiple farms. Their results showed predictive accuracy comparable to centralized approaches while maintaining local data confidentiality (Siniosoglou et al., 2023). Similarly, Kumar et al. (2022) introduced the PEFL architecture, combining perturbation encoding with federated LSTM to achieve accurate intrusion detection and data protection in smart farming, enhancing system robustness against adversarial threats (Kumar et al., 2022).

**Figure 12:**
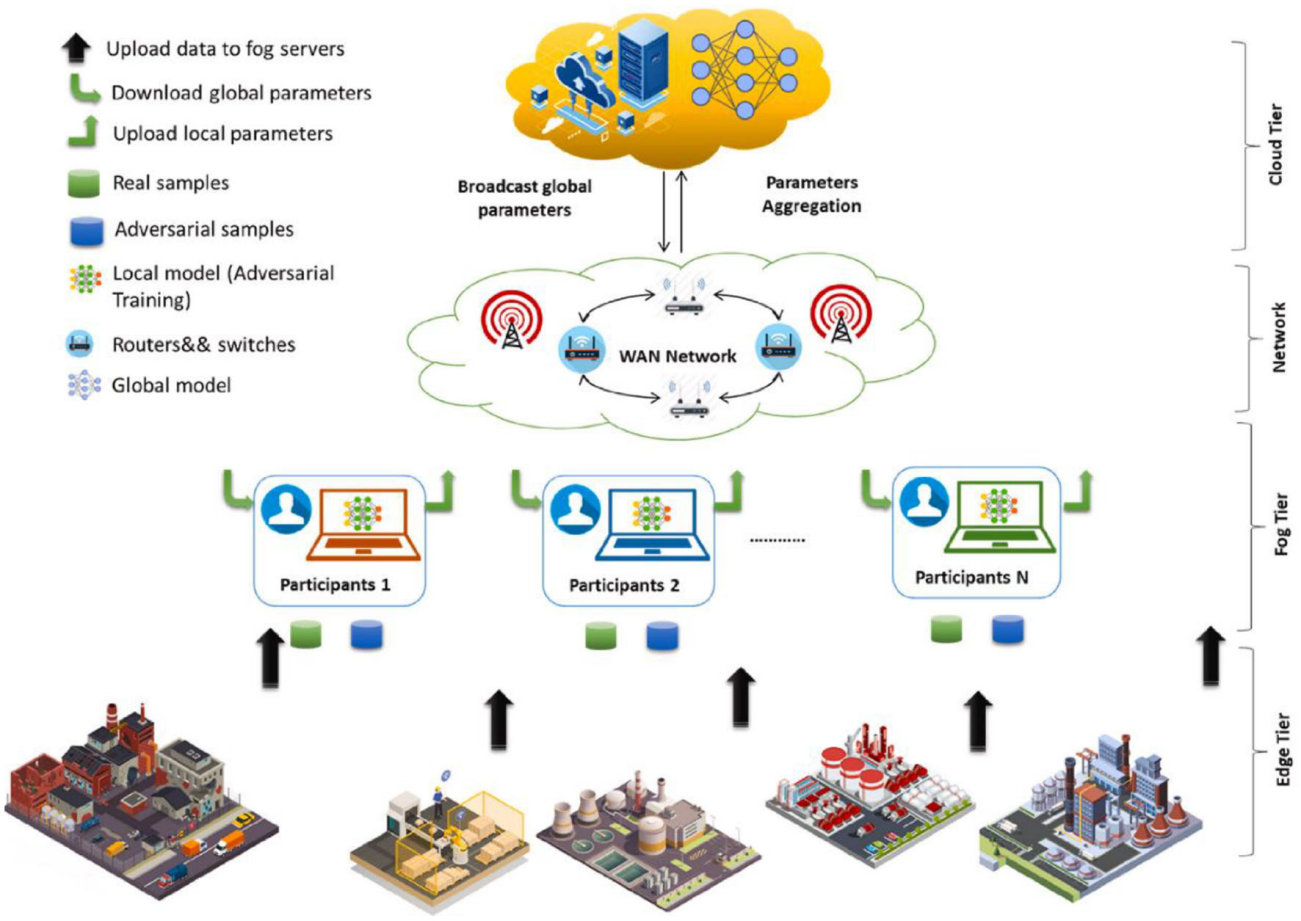
Illustration of the proposed privacy-preserving federated learning in Fog-assisted IoT. an ideal fog node for presuming a worldwide collector’s responsibility at each round (Abdel-Basset et al., 2022).

Looking forward, several critical directions for future research are as follows:

(1) Integration of Differential Privacy and Homomorphic Encryption: Developing hybrid schemes to defend against inference attacks on sensitive farm-level data during FL operations;
(2) Federated Incentive and Trust Mechanisms: Leveraging decentralized assessment and incentive protocols, potentially using blockchain, to encourage honest participation (Huang et al., 2023);
(3) Robust Modeling under Non-IID Data: Addressing statistical heterogeneity through frameworks such as PP-FDL, which improve model generalizability and adversarial resistance in federated settings (Abdel-Basset et al., 2022). As shown in **Figure 12**, the proposed fog computing-assisted privacy-preserving federated learning framework for the Internet of Things (IoT) dynamically selects an ideal fog node to act as the global aggregator.

### 6.5 Establishing a standardized framework for an open agricultural ecosystem

One of the major challenges currently facing smart agriculture systems is the lack of interoperability across platforms and the absence of unified standards. This leads to inefficient communication and collaboration between agricultural devices, sensor networks, and data platforms. Such “system silos” are particularly prominent in environments where devices from multiple vendors and heterogeneous communication protocols coexist. Building a standardized and open ecosystem is therefore not only a prerequisite for technical coordination but also the foundation for scalable industrial development.

Et-taibi et al. (2024) proposed a three-tier open-source architecture integrating IoT, cloud, and edge computing layers. Their approach advocates the use of open communication protocols, such as MQTT and CoAP, to facilitate multi-layer data exchange and interoperability among devices. This architecture significantly reduced deployment costs and improved data processing efficiency in smart agriculture systems (Et-taibi et al., 2024). Similarly, Trzec et al. (2022) designed a cloud-native microservices-based platform aimed at building an interoperable IoT ecosystem for agriculture. The platform flexibly integrates heterogeneous edge devices with data visualization services, enabling modular and dynamic configuration of agricultural monitoring systems (Tržec et al., 2022).

Kambala (2024) reviewed key challenges in cross-platform IoT gateway design for multi-domain integration. The study emphasized the critical role of middleware and standardized communication protocols—such as OPC-UA and MQTT—in addressing protocol heterogeneity, resource coordination, and secure data exchange. Furthermore, it highlighted the potential of blockchain and semantic ontology to enhance data trustworthiness and interoperability (Kambala, 2024).

Zhang et al. (2024) further noted the absence of unified standards in several core areas of the agricultural ecosystem, including sensor accuracy, data acquisition interfaces, and terminal software compatibility. The study called for self-governance mechanisms within the industry, supported by collaborative investment in information infrastructure. Moreover, it advocated for the specification of minimum standards for smart sensing devices, analytics software, and data interaction protocols (Zhang J. et al., 2024).

To address these challenges and foster an open, interoperable agricultural ecosystem, future research should focus on the following directions:

(1) Standardizing Interface Protocols and Semantic Data Models: Promote unified interface protocols and semantic frameworks such as the Smart Applications REFerence ontology (SAREF) for agricultural IoT devices;
(2) Establishing Conformance Testing Frameworks: Develop testing mechanisms to ensure consistency between edge control protocols and cloud interaction standards;
(3) Designing Secure Cross-Domain Trust Architectures: Construct trusted authentication, secure communication, and access control frameworks to guarantee secure collaboration across platforms;
(4) Encouraging Open Protocol Stacks and Testing Platforms: Facilitate rapid adoption by small- and medium-sized agricultural device vendors through community-supported open-source protocols and validation platforms.

### 6.6 Cyber-physical integration and real-time simulation enabled by digital twin technology

Digital Twin (DT) technology enables the construction of virtual models that are dynamically synchronized with real-world agricultural entities, including crop growth, soil moisture, climate conditions, and machinery status. As a key enabler of the paradigm shift from reactive to predictive decision-making in smart agriculture, DT systems—when supported by cloud–edge–device architectures—can execute real-time local inference at the edge, while utilizing cloud computing for large-scale modeling and policy optimization. This facilitates a tightly integrated control and simulation framework for precision agriculture.

**Figure 13:**
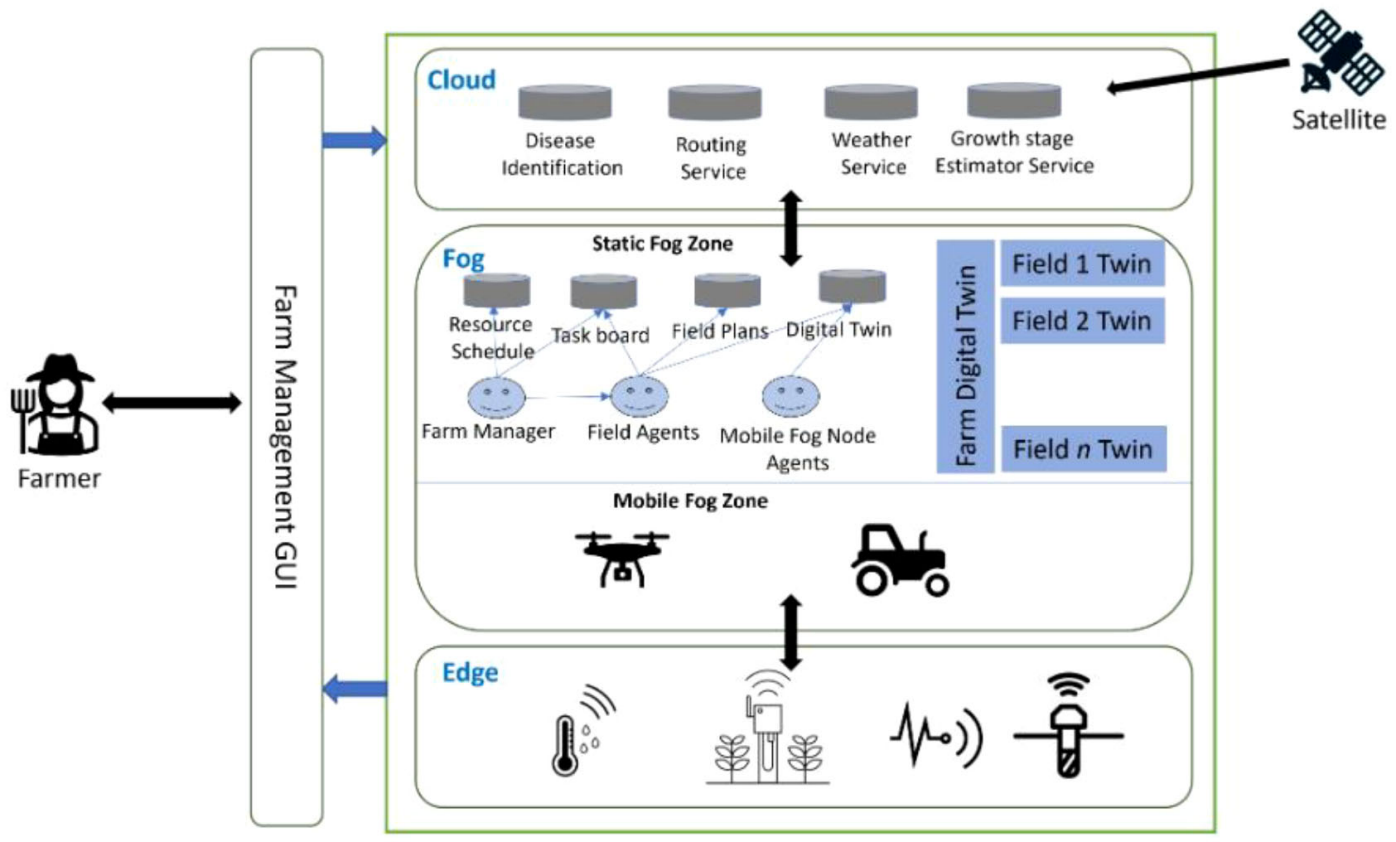
Proposed Architecture for Digital Twins in Smart Agriculture (Kalyani et al., 2024).

In recent years, the practical implementation of DTs in agriculture has advanced rapidly. Kalyani et al. (2024) proposed an agricultural DT model based on a cloud–fog–edge collaborative architecture, enabling contextualized scenario simulation for irrigation, fertilization, and pest control. Their model supports data-driven “what-if” simulations to assist decision-making (Kalyani et al., 2024). As illustrated in **Figure 13**, the proposed architecture for digital twins in smart agriculture integrates real-time data acquisition, edge computing, and cloud-based analytics to create virtual replicas of physical farming systems (Kalyani et al., 2024). Similarly, Peladarinos et al. (2023) developed a multi-source dynamic visualization platform that fuses high-resolution satellite imagery with sensor data from soil and weather networks, significantly improving farmers’ responsiveness to irrigation and fertilization decisions (Peladarinos et al., 2023).

In terms of system design, Zaķe and Majore (2022) introduced a multi-perspective modeling approach (MultiDigiII), which constructs coupled conceptual models at multiple levels of abstraction. This enhances the DT system’s capacity to represent and respond to complex agricultural phenomena such as climatic variability, pest outbreaks, and crop structural dynamics (Zaķe and Majore, 2022). Alves et al. (2023) developed an irrigation DT system integrating the FIWARE platform with real-time simulation tools, allowing multi-strategy irrigation testing and crop response prediction—providing both water-saving benefits and pre-deployment validation (Alves et al., 2023).

Moreover, Kim et al. (2023) proposed a high-frequency federated DT update mechanism tailored for agricultural IoT systems. Their system demonstrated excellent timeliness and adaptability in collaborative multi-farm simulations, offering early prediction capabilities for abrupt weather changes and device malfunctions (Kalyani and Collier, 2022).

To further enhance the effectiveness and scalability of DT applications in agriculture, future research should focus on the following directions:

(1) Semantic Integration with Agricultural Knowledge Bases: Combine DT systems with crop atlases, soil databases, and ontologies to achieve a synergy between knowledge-driven and data-driven simulation approaches;
(2) AI-Augmented Decision-Making: Incorporate deep reinforcement learning and multi-objective optimization to enable intelligent scheduling, environmental regulation, and disaster response;
(3) Standardized Modeling Frameworks: Develop domain-specific modeling languages and reusable component libraries to support cross-system deployment and interoperability of DT applications;
(4) Secure and Privacy-Preserving Architectures: Integrate homomorphic encryption and edge-level security modules to ensure data integrity, confidentiality, and trustworthiness within DT systems.

### 6.7 Integration of LLMs and AIGC in smart agriculture

In addition to the aforementioned directions, the emergence of large language models (LLMs) and AI-generated content (AIGC) offers novel opportunities for intelligent agricultural systems. LLMs, such as ChatGPT and other foundation models, can facilitate knowledge summarization, cross-lingual communication, advisory services, and context-aware decision support for farmers. AIGC technologies can automate report generation, assist in interpreting sensor data patterns, and produce real-time alerts or recommendations tailored to regional agronomic knowledge. These innovations are expected to further reduce the cognitive load on agricultural operators and improve decision-making agility in complex environments.

## 7 Conclusion

Cloud-Edge-Device collaborative computing significantly advances the capabilities of smart agricultural systems by enabling distributed intelligence, low latency, and optimized resource utilization. Through detailed examination of current implementations and technological architectures, this review highlights the profound potential of integrated computing layers to effectively manage complex agricultural environments. Despite substantial progress, several technical and practical challenges persist, notably in heterogeneous device management, data consistency, energy constraints, and security risks. Addressing these challenges will require multidisciplinary collaboration and continuous innovation in intelligent scheduling, lightweight edge AI algorithms, robust federated learning mechanisms, standardized system interoperability, and digital twin technologies. By proactively addressing these critical areas, the smart agriculture community can facilitate the transformation from reactive to predictive decision-making frameworks, driving sustainable agricultural practices and enhancing food security. Ultimately, the continued development and integration of Cloud-Edge-Device frameworks represent a strategic pathway toward realizing highly adaptive, efficient, and resilient agricultural systems worldwide.
